# The Early Response to Urea, Nitrate, or Ammonium and Iron Resupply in Tomato Roots Highlights the Induction of FER, bHLHs, UMAMITs, and MATEs

**DOI:** 10.1111/ppl.70977

**Published:** 2026-07-22

**Authors:** Arianna Lodovici, Leilei Zhang, Nicola Tomasi, Gabriella Vinci, Fabio Marroni, Barbara Piani, Fatemeh Hassanvand, Mustapha Arkoun, Luigi Lucini, Laura Zanin

**Affiliations:** ^1^ Department of Agricultural, Food, Environmental and Animal Sciences University of Udine Udine Italy; ^2^ Department for Sustainable Food Process, Research Centre for Nutrigenomics and Proteomics Università Cattolica del Sacro Cuore Piacenza Italy; ^3^ Plant Nutrition R&D Department Centre Mondial D'innovation of Roullier Group Saint‐Malo France

## Abstract

Depending on the nitrogen (N) form available to roots, plants activate different transcriptional and metabolic pathways; this can consequently translate into differences in the composition and release of root exudates. To address the existing knowledge gap regarding the relationship between the availability of Iron (Fe) and different N forms applied (nitrate, ammonium, or urea), the molecular mechanisms activated by plants to promote their acquisition have been investigated using omics data integration (“exudomic” × transcriptomic analyses). The transcriptomic and metabolomic analyses reveal a strong crosstalk among nutritional pathways, showing that tomato root responses to Fe resupply depend on the nitrogen form provided. Transcriptomic profiles after 4 h indicate similar responses under urea and ammonium, a trend also observed at the exudomic level after 24 h. Distinct molecular features emerged depending on the specific N form applied. In particular, Fe resupply with urea induced the upregulation of the expression of *FER* and *bHLH66*, moderately enhancing the Fe level in roots. Overall, integrated omics data highlight a complex network of transcription factors, metabolites, and transporters that may contribute to improved plant resilience under nutrient stress, such as transcription factors (bHLH, MYB, ZAT12), transporters (UMAMITs, MATEs) and a proteolytic regulator of a phenylpropanoid enzyme (KFB‐PAL). Within the framework of sustainable agriculture, this study corroborates the occurrence of a strong interplay between Fe‐ and N‐nutritional pathways and emphasizes how different N forms can modulate root exudation in the rhizosphere, thereby enhancing the uptake of other essential nutrients such as Fe.

## 1 Introduction

Iron (Fe) deficiency is considered to be one of the most important plant nutritional disorders as the bioavailability in soils of this element is limited (Lucena 2000; Briat et al. 2015; Liang 2022). In soils, Fe mainly occurs in poorly soluble Fe‐oxide, hydroxide, carbonate, silicate, and Fe‐phosphate minerals that scarcely contribute to the soluble fraction. More than 30% of the earth's arable land is calcareous (alkaline pH), a condition that greatly reduces Fe solubility in the soil and thus compromises the Fe bioavailability for plant nutrition (Nozoye et al. 2017; Ciurli et al. 2025; Khalil et al. 2025). Under Fe‐limiting conditions, nongrass species activate the so‐called *Strategy I*, a reduction‐based Fe‐acquisition mechanism, in which mobilization of the scarcely available Fe pools in soil is supported by acidification of the rhizosphere through proton extrusion, the release of root exudates, the reduction of chelated Fe(III) to the more soluble ferrous form (Fe^2+^), and the subsequent uptake of Fe^2+^ by root transporters. Root exudation plays an important role in this strategy, as plants secrete a variety of metabolites, including phenolic compounds, flavins, and organic acids, that can mobilize Fe from poorly soluble complexes and enhance its availability in the rhizosphere (Olsen et al. 1981; Hether et al. 1984; Astolfi et al. 2020). In contrast, grasses rely on the so‐called *Strategy II* mechanism, which involves the biosynthesis and root secretion of compounds belonging to the mugineic‐acid family (phytosiderophores, PS) in the rhizosphere, where they are able to chelate Fe. Iron is then taken up by the roots of grasses as Fe(III)–PS complex by transporters like YS1/YSL (Grillet and Schmidt 2019). Notably, components typical of one strategy have also been observed in crops primarily relying on the other Fe‐acquisition mechanism and vice versa, supporting a more flexible model in which Strategy I plants can also utilize Fe(III)‐chelating mechanisms similar to those of Strategy II (Zanin et al. 2017; Robe et al. 2025). The transcriptomic modulation in tomato roots under Fe deficiency has previously been characterized (Zamboni et al. 2012), as has that in other species. The Fe‐deficiency response in tomato is related to the modulation of a small set of genes, similarly to the so‐called Arabidopsis “ferrome” (Schmidt and Buckhout 2011; Gao and Dubos 2021; Riaz and Guerinot 2021).

In the past decades, the Fe‐deficiency response in plants, as well as the response to other nutritional deficiencies, has been separately investigated, but current findings have indicated that the plant response to multiple nutrient stresses cannot be inferred from a summation of the plant responses to individual stresses (for review, see Bouain et al. 2019). Indeed, changes in the bioavailability of one element affect the uptake of other elements in plants, causing alterations to sensing and signaling pathways dedicated to their acquisition (Rouached et al. 2010; Liang 2022; Vélez‐Bermúdez and Schmidt 2023). Among macronutrients, nitrogen (N) is the most commonly applied nutrient fertilizer, mainly in the form of urea, ammonium, or nitrate.

Regarding nitrate, the alkalinization of the rhizospheric solution (due to acquisition and assimilation of the anionic N form in root cells) limits Fe solubility and therefore it can be expected that nitrate nutrition may exacerbate Fe‐deficiency symptoms in plants. In sunflower, supplying nitrate led to alkalinization of leaf apoplastic pH and reduced the activity of ferric chelate reductase, generating visible symptoms of Fe chlorosis (Kosegarten et al. 1998, 1999; Mengel 1995). On the other hand, Fe is a cofactor (in the Fe–heme group or as part of Fe–S clusters) of enzymes involved in the reductive assimilatory pathway of nitrate, such as nitrate reductase (NR), nitrite reductase (NiR) and glutamate synthase (GOGAT; Rengel et al. 2022). Thus, under Fe deficiency, nitrate assimilation is usually slowed down in plants (Alcaraz et al. 1986; Borlotti et al. 2012) and triggers a reduction in net nitrate uptake into roots at the same time (Iacuzzo et al. 2011).

In contrast to nitrate, the ammonium nutrition (which is usually linked to an acidification of the root external media) increases the absorption of nutrients such as phosphorous, Fe, and manganese and impacts plant metabolism (Thomson et al. 1993; Sarasketa et al. 2016; De la Peña et al. 2022). Thus, especially in aerobic and calcareous soils, the synergistic effect of ammonium on the uptake of other nutrients can be positive for plant nutrition. In *Brachypodium distachyon* , investigation of ammonium nutrition determined a clear induction of the expression of genes responsive to Fe deficiency, such as those involved in the methionine salvage cycle and phytosiderophores (PS) synthesis (De la Peña et al. 2022). The upregulation of these pathways is extremely valuable for optimizing Fe acquisition in plants, especially in grasses, where the Fe uptake follows a PS chelation‐based strategy. Even the Fe translocation from roots to shoots seems to be promoted under ammonium nutrition as a consequence of the upregulation of *FRD3* (FERRIC REDUCTASE DEFECTIVE3) and *NAS1* (NICOTIANAMINE SYNTHASE1) genes, both involved in Fe translocation, leading to an increase in the soluble Fe levels in shoots and thus alleviating Fe‐deficiency symptoms (Zhu et al. 2019).

Despite urea being the most widely used N fertilizer worldwide, its interaction with Fe nutrition in plants remains poorly understood. Hsu and Ashmead (1984) reported that the foliar application of urea promoted Fe absorption, likely by increasing leaf permeability. In tomato, urea has been shown to induce metabolic reprogramming and alter micronutrient homeostasis, including secondary metabolite modulation and changes in N‐related compounds (Lodovici et al. 2024). Under Fe‐deficient conditions, the supply of Fe and urea induced an upregulation of FER in tomato roots with expression levels even higher than those recorded in Fe‐deficient plants or plants resupplied with Fe and inorganic N forms (Lodovici et al. 2024). Notably, a downregulation of the expression of *SlDUR3* (putatively coding for the urea transporter) was observed under Fe deficiency, suggesting that the two nutritional pathways are strongly connected (Lodovici et al. 2024).

The interplay between N‐forms, especially urea, and Fe nutrition is still poorly characterized, particularly regarding the role of different N sources in modulating plant response to low Fe availability (Zou et al. 2001; Nikolic et al. 2007; Borlotti et al. 2012; Liu et al. 2015; Chen et al. 2021). Based on the key role played by Fe in N assimilation and vice versa (i.e., changes in rhizospheric properties), it is reasonable to propose the occurrence of strong cross‐connections between the N‐ and Fe‐nutritional pathways and the close relationships in the regulation and activation of their reciprocal acquisition mechanisms (Liu et al. 2015; Chen et al. 2021). The study of the early responses to Fe resupply provides valuable insights into the mechanisms regulating Fe nutritional pathway. In the first hours after Fe repletion, rapid transcriptional changes occur before secondary physiological adjustments develop, while concurrent modifications in root exudation may reflect dynamic strategies for Fe mobilization, linking transcriptomic and exudomic responses. To address this, we examined the responsiveness of Fe‐deficient plants to Fe resupply under different N‐forms: urea, ammonium, or nitrate. The early response of tomato plants was evaluated after 4 h of N and Fe resupply, analyzing variations in the root transcriptomic profile and in the root exudomic composition. The latter was also evaluated after 24 h, as transcriptomic variations may result in physiological responses at later time.

## 2 Materials and Methods

### 2.1 Plant Growth Conditions

Tomato seeds ( *Solanum lycopersicum* L., cv “Marmande” from DOTTO Spa, Udine, Italy) were grown hydroponically as previously described by Lodovici et al. (2024). Thirty‐five‐day‐old plants were transferred to an N‐free and Fe‐free nutrient solution (mM: 0.7 K_2_SO_4_, 0.1 KCl, 1.0 CaSO_4_, 0.5 MgSO_4_, 0.1 KH_2_PO_4_; μM: 10 H_3_BO_3_, 0.50 MnSO_4_, 0.50 ZnSO_4_, 0.20 CuSO_4_, 0.07 Na_2_MoO_4_), and under these conditions, plants were grown for 7 days (renewed every 3 days; 1.5 mM MES‐BTP pH 6.0).

**FIGURE 1:**
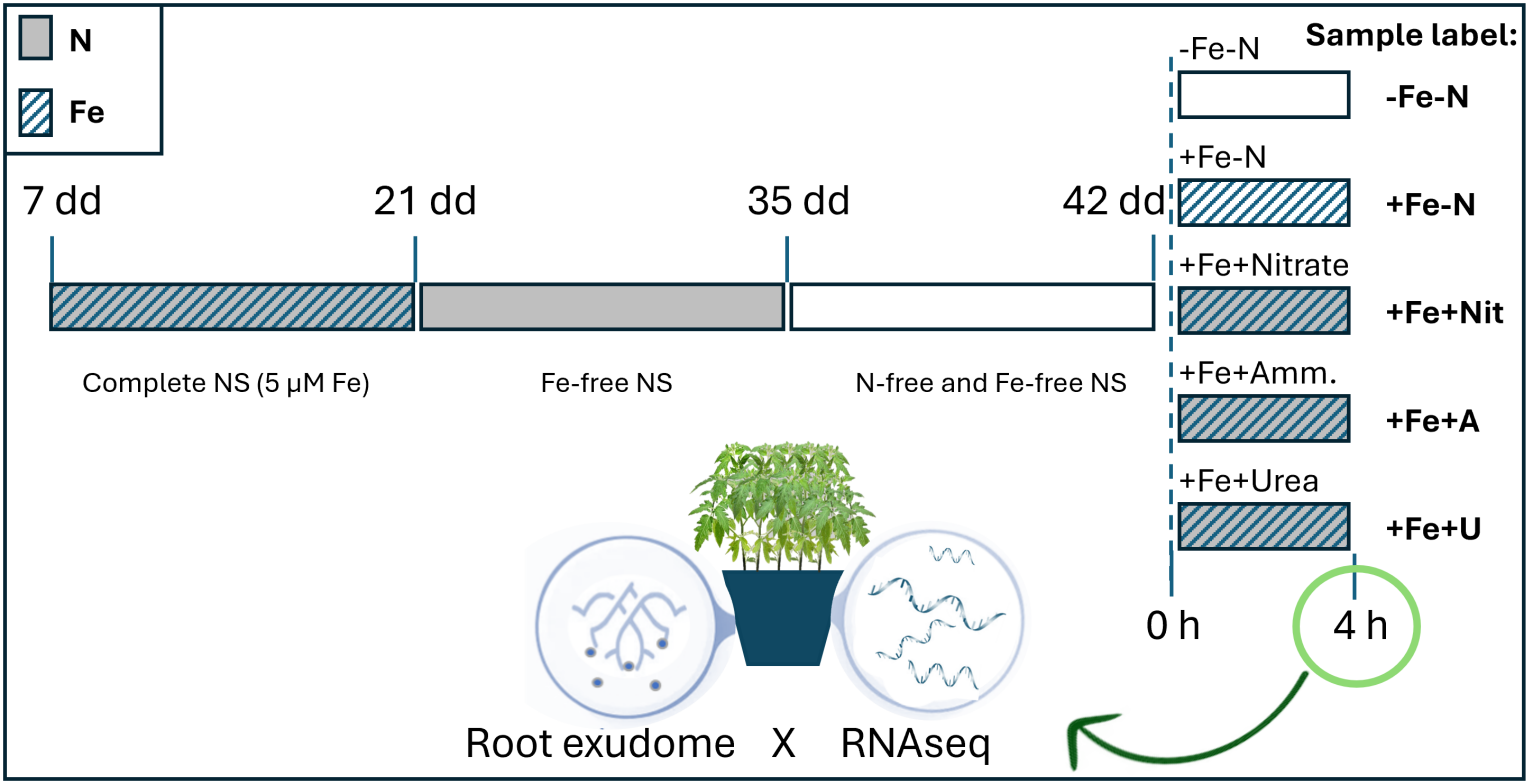
Experimental setup scheme. Tomato plants (42‐d‐o) grown under Fe deficiency and N depletion were exposed to nutrient solution where Fe and N were resupplied at a final concentration of 5 μM Fe and 2 mM total N (applied in form of nitrate, urea or ammonium: +Fe + Nit, +Fe + U, +Fe + A). After 4 h variations in the root transcriptomic profiles and root exudates were evaluated. The root exudate composition was also evaluated after 24 h.

Forty‐two‐day‐old plants were exposed up to 24 h to the previously described nutrient solution supplied with Fe (5 μM Fe supplied as Fe‐EDTA) and different N forms (2 mM total N supplied as potassium nitrate, ammonium sulfate, or urea) as follows: 5 μM Fe‐EDTA and 2 mM KNO_3_ (+Fe + Nit treatment), 5 μM Fe‐EDTA and 1 mM (NH_4_)_2_SO_4_ (+Fe + A treatment), 5 μM Fe‐EDTA and 1 mM CH_4_N_2_O (+Fe + U treatment). As controls, plants were maintained in Fe‐free and N‐free nutrient solution (−Fe–N treatment) or supplied with 5 μM Fe (supplied as Fe‐EDTA and maintained in N‐free nutrient solution, +Fe–N treatment, Figure 1). For the metabolomic studies, an additional condition was used as reference: 42‐day‐old plants grown under Fe sufficiency, described by Lodovici et al. (2024), were used (100 μM Fe continuously supplied as Fe‐EDTA to the previously described nutrient solution, +Fe/+Fe–N treatment).

During the whole growing period, the controlled climatic conditions were the following: 16/8 (day/night) photoperiod; 220 μmol m^−2^ s^−1^ light intensity; 25/20°C (day/night) temperature, and 70%–80% relative humidity. The Fe‐induced chlorosis level was monitored using a SPAD instrument (SPAD‐502, Minolta).

For root transcriptomic analyses: after 4 h of treatment (treatment started after 1 h of the onset of the light phase), each treatment with three biological replicates (roots from four plants pooled together) was collected, immediately frozen in liquid N_2,_ and stored at −80°C until subsequent molecular work.

For root exudomic analyses (metabolomic analyses of root exudates): after 4 and 24 h, for each treatment, four biological replicates of root exudates were collected. For each biological replicate, roots of four intact tomato plants were submerged in 50 mL of 0.5 mM CaSO_4_ solution for 2 h in sterile plastic bags, then the exudation solution was filtered (PTFE Syringe Filters, 0.45 μm), and stored at −20°C until further processing.

### 2.2 Transcriptomic Analyses

One gram of root tissue was homogenized in liquid N_2_, and total RNA was isolated from approximately 60 mg of the resulting powder using the Spectrum Plant Total RNA Kit (Sigma‐Aldrich) following the manufacturer's instructions. To ensure no genomic DNA contamination, 1 μg of total RNA was electrophoretically analyzed on a 1% agarose gel. RNA concentration and integrity were assessed using the Qubit 2.0 Fluorometer (Life Technologies) and the Agilent 2100 Bioanalyzer system, according to the manufacturer's protocols (Agilent Technologies). The preparation of the cDNA library and subsequent RNA sequencing were carried out by IGA Technology Services s.r.l. (Udine). Library preparation followed the Illumina TrueSeq 2.0 protocol, using 2 μg of total RNA per sample (Venuti et al. 2019). Libraries were prepared using the Universal Plus mRNA‐Seq kit (Tecan Genomics) according to the manufacturer's instructions. Libraries were then sequenced on paired‐end 150 bp mode on NovaSeq 6000 (Illumina). Adapters were masked by the service provider. The obtained reads were aligned to the SL4.0 reference genome and ITAG4.0 transcriptome (Hosmani et al. 2019) using hisat2 (Kim et al. 2019) with default settings.

### 2.3 Metabolomic Analysis of Root Exudates

The exudate solutions were freeze‐dried and then resuspended into 1 mL of ultra‐pure water and the exudate profiling was conducted using a 6560‐drift tube‐ion mobility‐quadrupole‐time of flight‐high resolution mass spectrometer (DTIM‐UHPLC‐QTOF‐HRMS; Agilent Technologies) with an injection volume of 19 μL, as detailly described in Supporting Information. Data annotation of raw mass features was processed using the software Profinder B.07 (Agilent Technologies), based on the “find‐by‐formula” algorithm against a homemade database (based on literature information), reaching a confidence Level 2 of identification (i.e., putatively annotated compounds, COSMOS standards in metabolomics) in according with Metabolomics Standards Initiative (MSI). The annotated features were aligned with an accuracy of 5 ppm and a retention time shift of 0.05 min. Afterward, the isotopic pattern of molecular features, including accurate monoisotopic mass, isotope spacing, and ratio, was used for annotation, following the method described in Supporting Information.

For the detection of the organic acids, the filtered root exudates were thawed and transferred into an autosampler vial. Twenty microliters were injected into HPLC, which included a Shimadzu LC‐20AT pump, a vacuum degasser, a Prominence SPD‐M20A photodiode array detector (set at 220 nm), a Prominence SIL‐20AC HT autosampler and a Prominence CTO‐20 AC column oven set at 40°C (Shimadzu Corporation). The HPLC separation was achieved using a cation exchange column (Aminex HPX‐87H; 300 × 7.8 mm, Biorad) and a precolumn. Equipment control, data acquisition and integration were performed with Shimadzu LabSolutions (Ver. 5.54 SP2) software. An isocratic program was run with 8 mM H_2_SO_4_ as a mobile phase at a flow rate of 0.6 mL min^−1^. Peaks of acids were identified by comparing retention times of standards, obtained after appropriate dilution of the reference standards with H_2_SO_4_ 8 mM, to those of the samples, and quantification was based on peak area measurements by the external standard method. Standards of pyruvic, malic, fumaric, succinic, lactic, acetic, oxalic acid were purchased from Sigma‐Aldrich, citric acid was from Carlo Erba Reagents S.r.l.

### 2.4 Transcriptomic and Metabolomic Data Integration

Metabolomic data from tomato root exudates (4 and 24 h) were integrated with root transcriptomic data (4 h) using Data Integration Analysis for Biomarker discovery using Latent variable approaches for Omics studies (DIABLO) framework was implemented within the “mixOmics” R package (version 6.22). The DIABLO model was optimized using the framework's tuning function, identifying an optimum of three components by minimizing the balanced error rate related to centroid distances. Afterward, significant features contributing to the differentiation between treatments were identified for each component, e.g., 90, 7, and 20 for Component 1; 90, 20, and 10 for Component 2; 70, 30, and 7 for Component 3, as transcriptomics, 4 h exudates and 24 h exudates, respectively. The results for the optimized DIABLO model were visualized through block contribution plots for each dataset (“T” for transcriptomics, “ID4” for exudates 4 h, and “ID” for exudates 24 h). The highly correlated features (key biomarkers; *r* > |0.9|) deriving from the three blocks were used to build network analysis using the “igraph” R package.

### 2.5 Statistical Analyses

**FIGURE 2:**
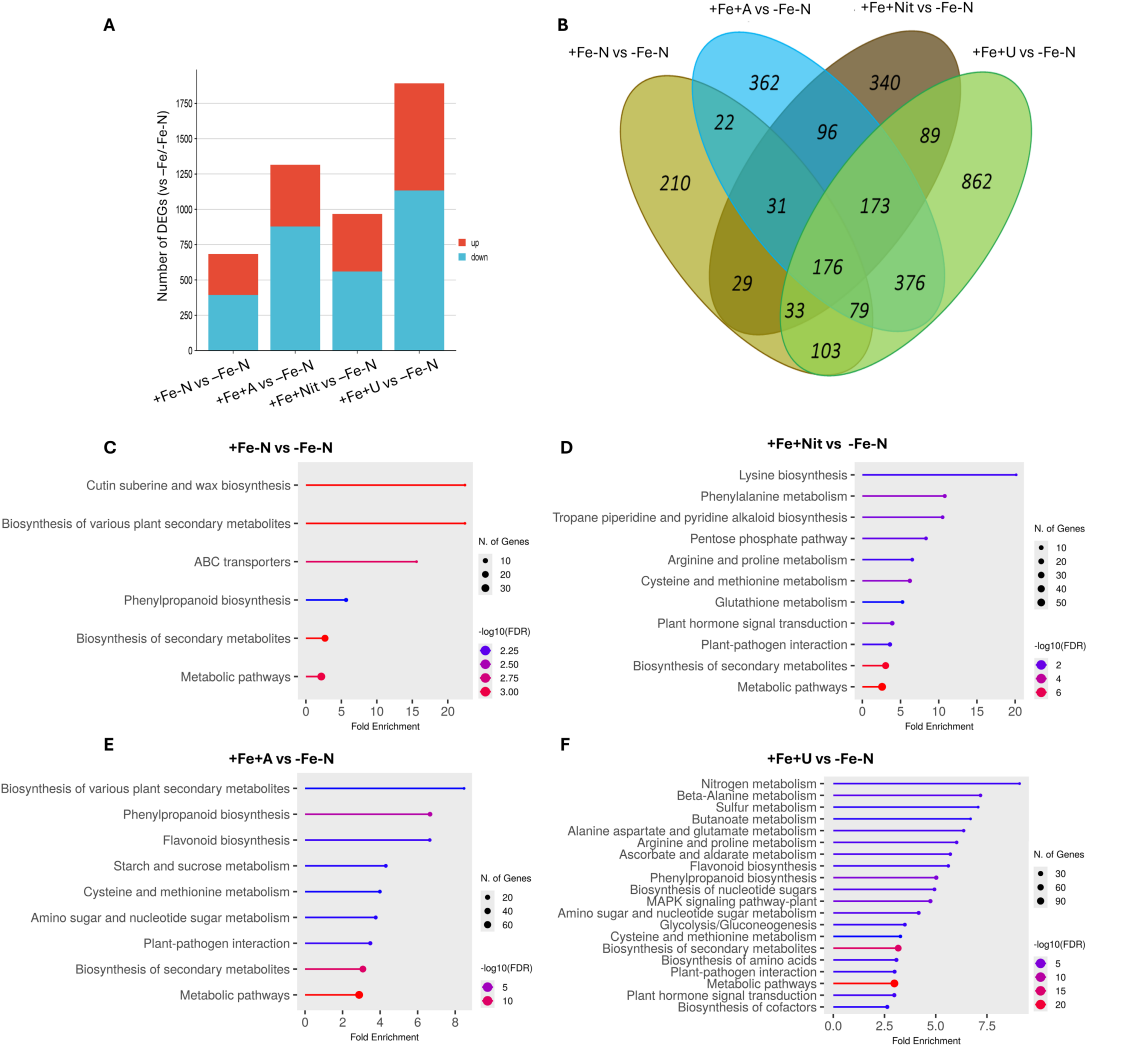
Number of differential expressed genes (DEGs, A); Venn diagram of DEGs modulated by +Fe–N, +Fe + A, +Fe + Nit, +Fe + U in comparison to −Fe–N (cutoff: |Log_2_FC| ≥ 1.00, *N* = 3, *q* ≤ 0.05) (B); KEGG enrichment analyses of DEGs modulated by +Fe–N, +Fe + A, +Fe + Nit, +Fe + U in comparison to −Fe–N (C–F).

Regarding the transcriptomic analyses, the differential expression was analyzed with cuffdiff (Trapnell et al. 2012) using default parameters, including the geometric method for library size normalization (Anders and Huber 2010). To reduce false positives, the FDR‐corrected *p*‐value (*q*‐value) was used to assess significance. The 15 FPKM datasets are visualized as clustering and heatmap in Figure S1A. Five transcriptomic profiles (−Fe–N, +Fe–N, +Fe + Nit, +Fe + U, +Fe + A) were generated by averaging expression across replicates (Figure S1B–E). The list of differentially expressed genes divided accordingly to Venn diagram regions is provided as Table S1 (vs −Fe–N; |Log_2_FC| ≥ 1.00, *N* = 3, *q* ≤ 0.05). Functional annotation of the reconstructed transcriptome utilized gene ontology (Ashburner et al. 2000) and KEGG (Kanehisa et al. 2016). Gene ontology (GO) analysis and enrichment were conducted using the singular enrichment analysis (SEA) of AgriGO v2.0 (Tian et al. 2017). Enrichment was determined using hypergeometric tests with Yekutieli adjustment for multiple comparisons (*p* ≤ 0.05) (Table S2 and Figures S2 and S3). The transcriptomic modulation was also visualized using ShinyGo 0.82 (https://bioinformatics.sdstate.edu/go/; Ge et al. 2020; Figure 2), SRplot (https://www.bioinformatics.com.cn/; Tang et al. 2023, Figure S1).

Regarding the analyses of root exudates, raw data underwent Log_2_ transformation, 75th percentile normalization, and baseline correction against each compound's median using Mass Profiler Professional 12.6 (Agilent Technologies). Under each collection time point (exudate collected after 4 and 24 h), the ANOVA (*α* = 0.05) statistical analysis was carried out to identify significant metabolites modulated by treatments and compare them to the control −Fe–N. Then, fold change analysis (expressed as Log_2_FC) was conducted to investigate the modulation of significant metabolites.

To investigate the overall pattern of distribution driven by different treatments, unsupervised hierarchical cluster analysis (HCA) was conducted. It employed Euclidean distance and Ward's linkage method to cluster separately exudates collected after 4 and 24 h treatment. Furthermore, the supervised orthogonal projection to latent structures discriminant analysis (OPLS‐DA), using the SIMCA software (v.16, Umetrics), was carried out separately for exudates after 4 and 24 h to better identify Variable Importance in Projection (VIP) markers associated with different treatments compared with the control −Fe–N. The VIP metabolites with a score > 1 were selected to build a bar plot representing the classes of metabolites that are highly discriminant, together with their Log_2_FC regulation, calculated from the pair comparison between treatments vs. control −Fe–N.

## 3 Results

### 3.1 Root Transcriptomics

Transcriptomic analyses were performed on Fe‐deficient tomato roots after 4 h of treatment (no resupply (−Fe–N) or resupply of Fe in combination with different N forms: either nitrate, urea, ammonium or without N (+Fe + Nit, +Fe + U, +Fe + A, +Fe–N)). The clustering analysis indicated greater similarity between the transcriptomic profiles of no‐resupplied plants (−Fe–N) and plants resupplied with only Fe (+Fe–N) than with those of the other treatments, where Fe was resupplied along with N (+Fe + Nit, +Fe + U, +Fe + A; Figure S1).

Differential expression analyses indicated that in comparison to control plants (−Fe–N), the Fe resupply induced the modulation of 683 Differentially Expressed Genes (DEGs; 288 upregulated and 395 downregulated by +Fe–N vs. −Fe–N); whereas the resupply of Fe and N (in form of nitrate, urea or ammonium) induced 967, 1891, or 1315 DEGs in roots (+Fe + Nit, +Fe + U, +Fe + A vs. −Fe–N, respectively, Log_2_|FC| ≥ 1.00, *N* = 3, *q* ≤ 0.05; Table 1 and Figure 2A,B).

**TABLE 1:** RNAseq results: Number of all the DEGs significantly modulated in seven comparisons (cutoff: |Log_2_FC| ≥ 1.00, *N* = 3, *q* ≤ 0.05).

Comparison	Up	Down	Total
+Fe + Nit vs. −Fe–N	406	561	967
+Fe + U vs. −Fe–N	757	1134	1891
+Fe + A vs. −Fe–N	436	879	1315
+Fe–N vs. −Fe–N	288	395	683
+Fe + Nit vs. + Fe–N	428	328	756
+Fe + U vs. + Fe–N	527	549	1076
+Fe + A vs. + Fe–N	285	441	726

**FIGURE 3:**
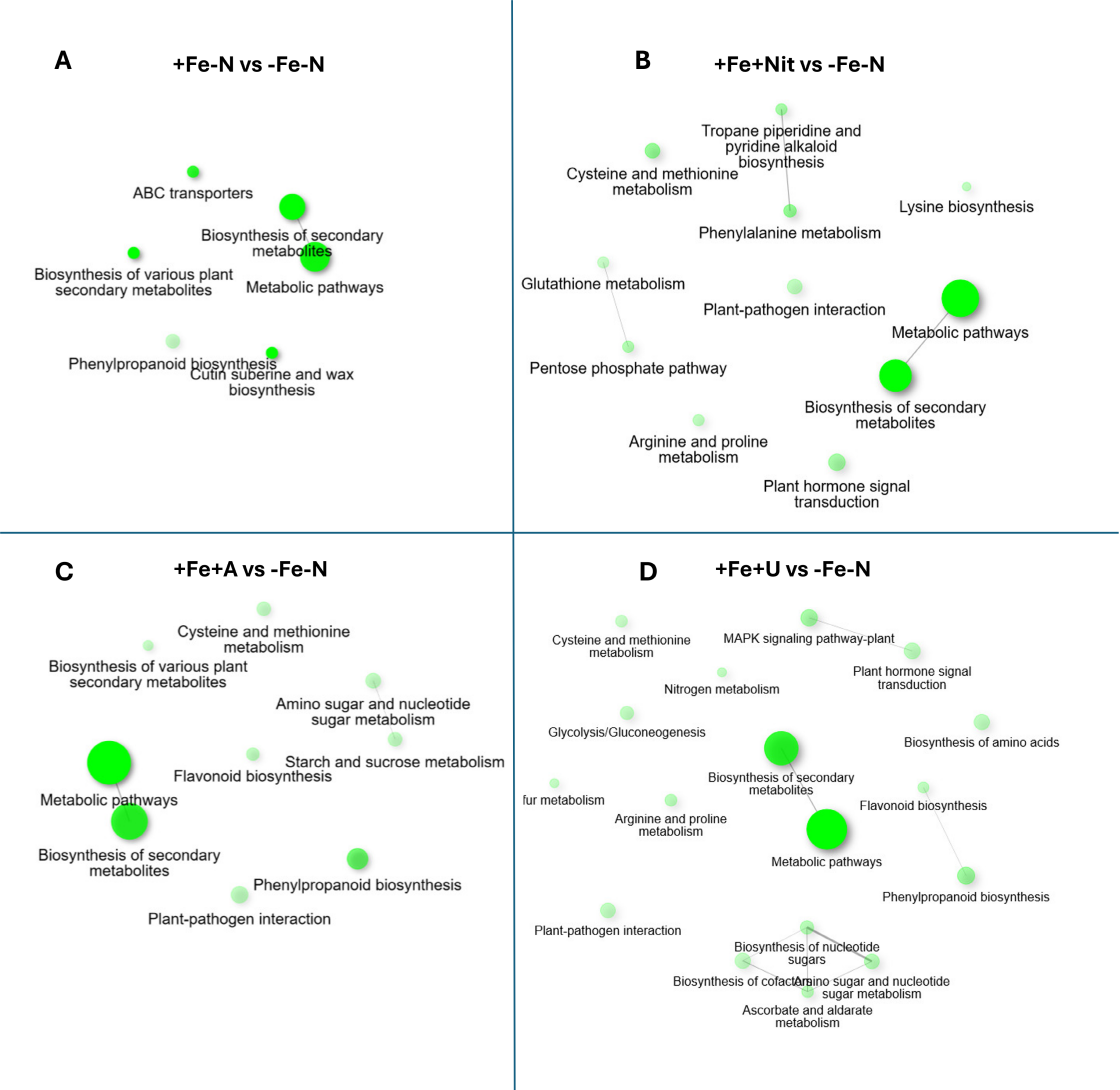
Networks showing the relationships between KEGG‐enriched pathways, based on KEGG enrichment analyses for the comparisons: +Fe–N vs. −Fe–N (A), +Fe + Nit vs. −Fe–N (B), +Fe + A vs. −Fe–N (C), +Fe + U vs. −Fe–N (D). Two pathways (nodes) are connected if they share 20% (default) or more genes. Darker nodes are more significantly enriched gene sets. Bigger nodes represent larger gene sets. Thicker edges represent more overlapped genes.

The functional analyses of DEGs, and, in particular, the KEGG enrichment analyses, indicated that the biosynthesis of the secondary metabolite pathway was modulated by all treatments. Moreover, compared to the others, the Fe and urea treatment induced the modulation of a larger number of pathways in roots. These pathways were mainly related to N and sulfur metabolism, flavonoid biosynthesis, amino acid and nucleotide sugar biosynthesis, cofactor metabolism, as well as hormone‐ and MAPK‐signaling transduction pathways. A smaller number of pathways were modulated by ammonium treatment, most of which overlapped with those modulated by the combined Fe and urea treatment (+Fe + U), such as flavonoid biosynthesis and amino sugar and nucleotide sugar metabolisms. The response induced by Fe and nitrate was characterized by a demand for reducing power and oxidative stress management, as indicated by modulation of the pentose phosphate pathway, glutathione metabolism, methionine and cysteine metabolisms, phenylalanine metabolism, lysine biosynthesis, and tropane, piperidine, and pyridine alkaloid biosynthesis, among others. The pathways modulated by Fe alone (+Fe–N) were mainly related to cell wall permeability and transmembrane transport (cutin, suberin, and wax biosynthesis and ABC transporters, Figures 2 and 3). In agreement with the KEGG enrichment, the GO analyses indicated a significant enrichment of transcripts involved in antioxidant activity and transporter activity; the GO enrichment analyses are provided as Supporting Information (Figures S2 and S3 and Table S2). The GO classes referred to cellular components, a high number of modulated transcripts are referred to as coded proteins having a membrane or extracellular localization. In particular, the +Fe + U treatment leads to the highest number of transcripts coding for membrane‐localized proteins in comparison to the other N forms, ammonium or nitrate (Figure S2).

**FIGURE 4:**
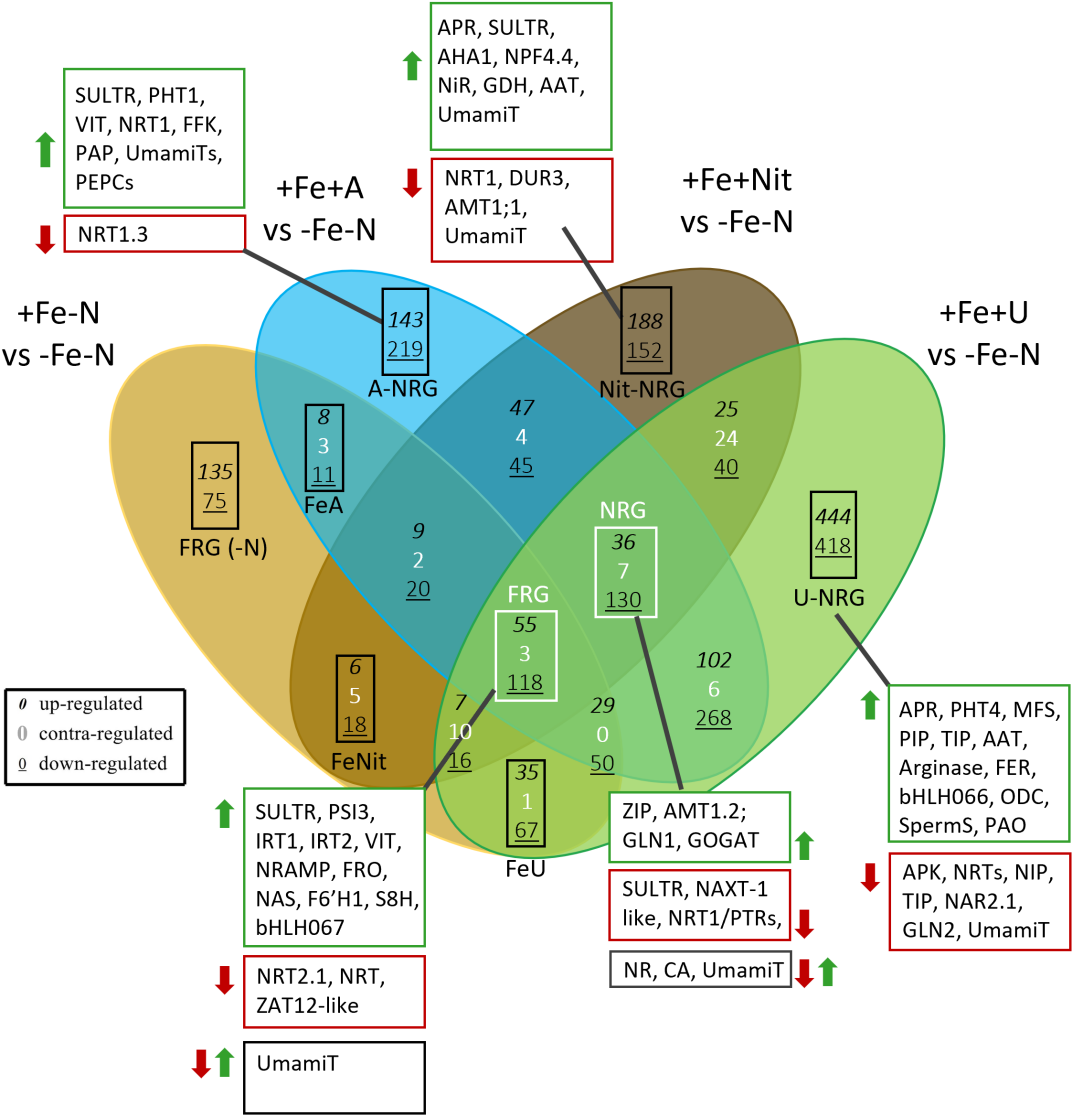
Venn diagram of DEGs in comparison to −Fe–N (|Log_2_FC| ≥ 1.00, *N* = 3, *q* ≤ 0.05). The upregulated significant DEGs are shown in italic black bold numbers, the downregulated DEGs are shown as underlined black numbers, the contra‐regulated DEGs are indicated in white. The names of DEGs presented in the discussion are provided within boxes (see Table S1 for the complete list of DEGs).

To visualize the transcriptional modulation, the names of key genes related to N and Fe nutrition have been indicated in the Venn diagram's regions (Figure 4, the complete list of significant DEGs divided by the Venn diagram's regions is reported as in Table S1). The Venn diagram shows that 176 DEGs were commonly modulated by all four comparisons mentioned above (+Fe–N, +Fe + Nit, +Fe + U, +Fe + A vs. + Fe–N): 55 upregulated DEGs, 118 downregulated ones, and three contra‐regulated by all four comparisons; these modulations are ascribable to the tomato response to Fe, independently to the N availability (hereafter called Fe‐Responsive Genes, FRGs, Figure 4). Moreover, the Venn diagram highlighted that +Fe + Nit, +Fe + U, +Fe + A vs. −Fe–N all modulated 173 DEGs (36 upregulated, 130 downregulated, and seven contra‐regulated transcripts) that were not shared by the comparison +Fe–N vs. −Fe–N; these modulations can be assumed to be related more to the N addition than to the Fe supply (hereafter it is referred as N‐Responsive Genes, NRGs, Figure 4).

Regarding FRG region, some genes known to be responsive in the short term to the Fe resupply were modulated, in particular some known Strategy‐I genes were induced by Fe resupply (e.g., *IRT1*, *IRT2*, *VIT*, *NRAMP*, *FRO*, *NAS*, *F6′H1*, *S8H*, *SULTR*, *PSI3*, *CYP82C4*), in particular two *VTLs*, *IRT2* (*Solyc02g069190*), *FRO2* (*Solyc01g094910*) and a *F6′H1* (*Solyc10g032565*) were upregulated between 6.5‐ and 47‐fold in each comparison (Table S8). Whereas some genes coding for nitrate transporters (NRTs) were downregulated (Table S1), in particular, in urea and ammonium treatment, where an *anion transporter (NRT1/PTR)* is strongly downregulated (more than 10×; Table S7). The modulation of the NRG region was related to transport and assimilation of ammonium (AMT, GS, GOGAT), whereas nitrate efflux transporter (NAXT) and other transporters were downregulated.

The Venn diagram highlighted that some DEGs were specifically responsive depending on the N‐form applied: 340 DEGs were specifically responsive to nitrate (region *Nitrate N‐Responsive Genes*, Nit‐NRG), 862 were responsive to urea (region *Urea N‐Responsive Genes*, U‐NRG), and 362 to ammonium (region *Ammonium N‐Responsive Genes*, A‐NRG; Figure 4). The regions FeNit, FeU, and FeA refer to those transcripts (29, 103, and 22 DEGs, respectively) that were modulated by Fe when applied in combination with nitrate or urea or ammonium, respectively (Figure 4 and Table S1). The modulation of 210 DEGs, FRG(–N), was induced only by Fe resupply in N‐deficient condition, as it was not shared by the other treatments where Fe was applied in combination with N. Among the 15 most upregulated genes by Fe resupply alone two *VTL* genes and genes encoding enzymes involved in the biosynthesis of phenylpropanoids were identified (*feruloyl‐CoA 6 hydroxylase*, *scopoletin 8‐hydroxylase*, *flavin monooxygenase*; Table S7). About those genes most repressed, mainly transcription factors (four DREBs, two ERFs, one MYB) and one NRT1 were found.

### 3.2 Root Exudates Metabolomics

Root exudates of tomato plants were collected after 4 and 24 h of the Fe and N resupply and compared to the control plants (not resupplied plants, −Fe–N) referred to the relative collecting time (4 or 24 h). The untargeted metabolomics approach was employed to conduct a comprehensive investigation of exudated metabolites, allowing us to identify 341 and 357 annotated features for 4 (Table S4A) and 24 h (Table S4B), respectively. The pattern of metabolite distribution and modulation among different treatments was inspected through HCA (Figure S6), specifically reporting exudates collected after 4 (Figure S6A) and 24 h (Figure S6B) of the Fe and N resupply. The tomato exudate profile after 4 h reported two different clusters. The first one was represented by the resupply of Fe and N under ammonium (+Fe + A) and nitrate forms (+Fe + Nit). The second cluster represented the similarities between the control −Fe–N with +Fe–N and +Fe + U. Regarding the exudate profile after 24 h, the most significant difference observed was the treatment represented by +Fe + Nit, which clustered and separated from all other groups. Among other treatments, the +Fe–N and −Fe–N groups and +Fe + U and +Fe + A groups were clustered in two main subclusters.

**FIGURE 5:**
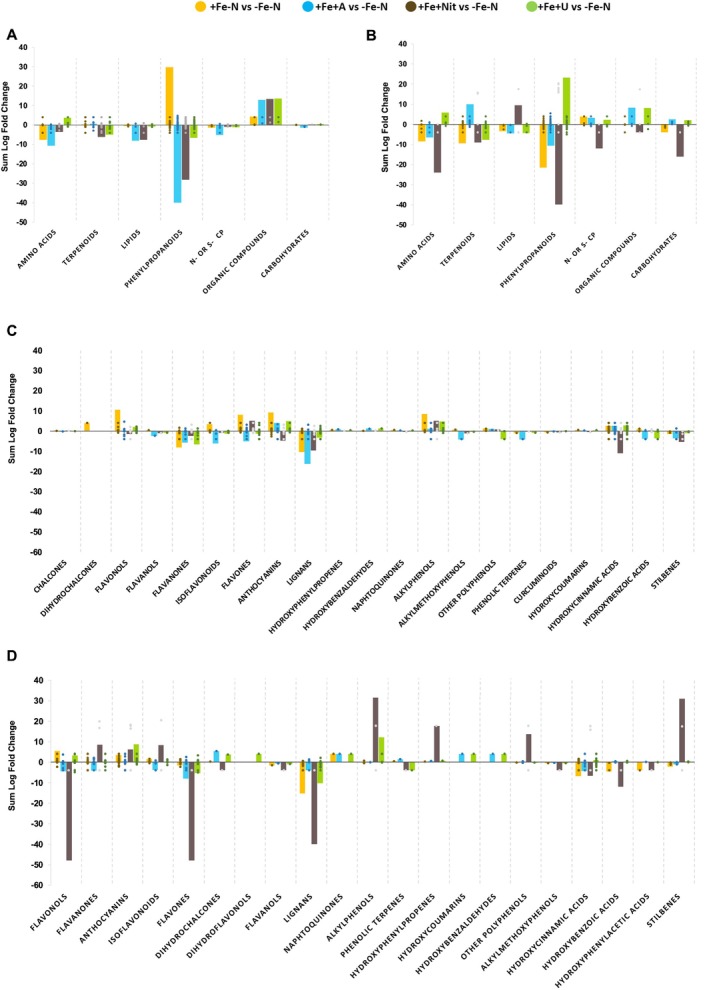
Classification of the modulated compounds into metabolic classes after unsupervised OPLS‐DA analysis. The analyses of the root exudates after 4 h refer to (A) and (C), whereas the analyses of the root exudates after 24 h refer to (B) and (D). The bars refer to the sum of Log_2_FC metabolites in the class, whereas the dots represent the distribution of the individual Log_2_FC metabolites in the classes. Color legend: Yellow bars refer to +Fe–N in comparison to −Fe–N, light blue +Fe + A, brown +Fe + Nit, light green +Fe + U, respectively.

Furthermore, supervised OPLS‐DA models were used to compare treatments with the control, aiming to identify biomarkers specifically associated with each treatment. The quality of OPLS‐DA models was further assessed based on goodness‐of‐fit (*R* ^2^ *Y*) and goodness‐of‐prediction (*Q* ^2^ *Y*). Specifically, metabolites with the strongest influence in the discrimination between treatments, as determined by a VIP score threshold of 1, were classified into compound classes and represented in Figure 5. These results are presented for exudates at 4 h (Figure 5A and Table S4C) and 24 h (Figure 5B and Table S4D).

At 4 h after Fe and N resupply, the main class of exudate metabolites was represented by phenylpropanoids and organic compounds (as detailed in Figure 5A and Table S4C). Specifically, Fe resupply (+Fe–N) led to an increased accumulation of phenylpropanoids, primarily flavonols, anthocyanins, flavones, and isoflavonoids (Figure 5C). Conversely, the simultaneous resupply of both Fe and N resulted in an overall downregulation of terpenoids, phenylpropanoids, and N‐ and sulfur‐containing compounds (Figure 5A).

The metabolite classes modulated after 24 h of Fe and N resupply are presented in Figure 5B (and Table S4D). In general, amino acids and terpenoids exhibited a similar regulatory trend to that observed in the exudate profile at 4 h after Fe and N resupply, with a more pronounced modulation at the later time point. Concerning other classes of exudate metabolites, the treatment +Fe + Nit showed the highest degree of modulation in the exudate profile, specifically leading to the accumulation of lipids, while inducing a reduction in phenylpropanoids, nitrogen‐ and sulfur‐containing compounds, organic compounds, and carbohydrates compared to the control. The most affected class was phenylpropanoids, including flavonols, flavones, and lignans (Figure 5D and Table S4D). Nevertheless, the +Fe + Nit condition also produced an increased accumulation of alkylphenols, isoflavonoids, stilbenes, and other low molecular weight phenolics, which all belong to the class of phenylpropanoids (Figure 5D and Table S4D).

Finally, an ANOVA was performed on the overall exudate metabolite profile at 4 and 24 h following Fe and N resupply to identify metabolites significantly influenced by the treatments. At 4 h posttreatment, 10, 6, 10, and 9 differentially modulated metabolites were identified in the root exudates of +Fe + A, +Fe + Nit, +Fe + U, +Fe–N plants, respectively, compared to control plants (−Fe–N, Table S5). After 24 h, the number of differentially modulated metabolites in the root exudates was 6, 5, 4, and 5 for +Fe + A, +Fe + Nit, +Fe + U, +Fe–N plants, respectively, in comparison to control plants (−Fe–N, Table S5).

Regarding the organic acids, the HPLC analyses on fresh samples of root exudates allowed the detection in all samples of citric, malic, fumaric, and succinic acids. However, no significant changes in the organic composition were observed depending on the N form applied along with Fe (among −Fe–N, +Fe–N, +Fe + Nit, +Fe + A, +Fe + U, Figure S7).

### 3.3 Transcriptomic and Metabolomic Data Integration

**FIGURE 6:**
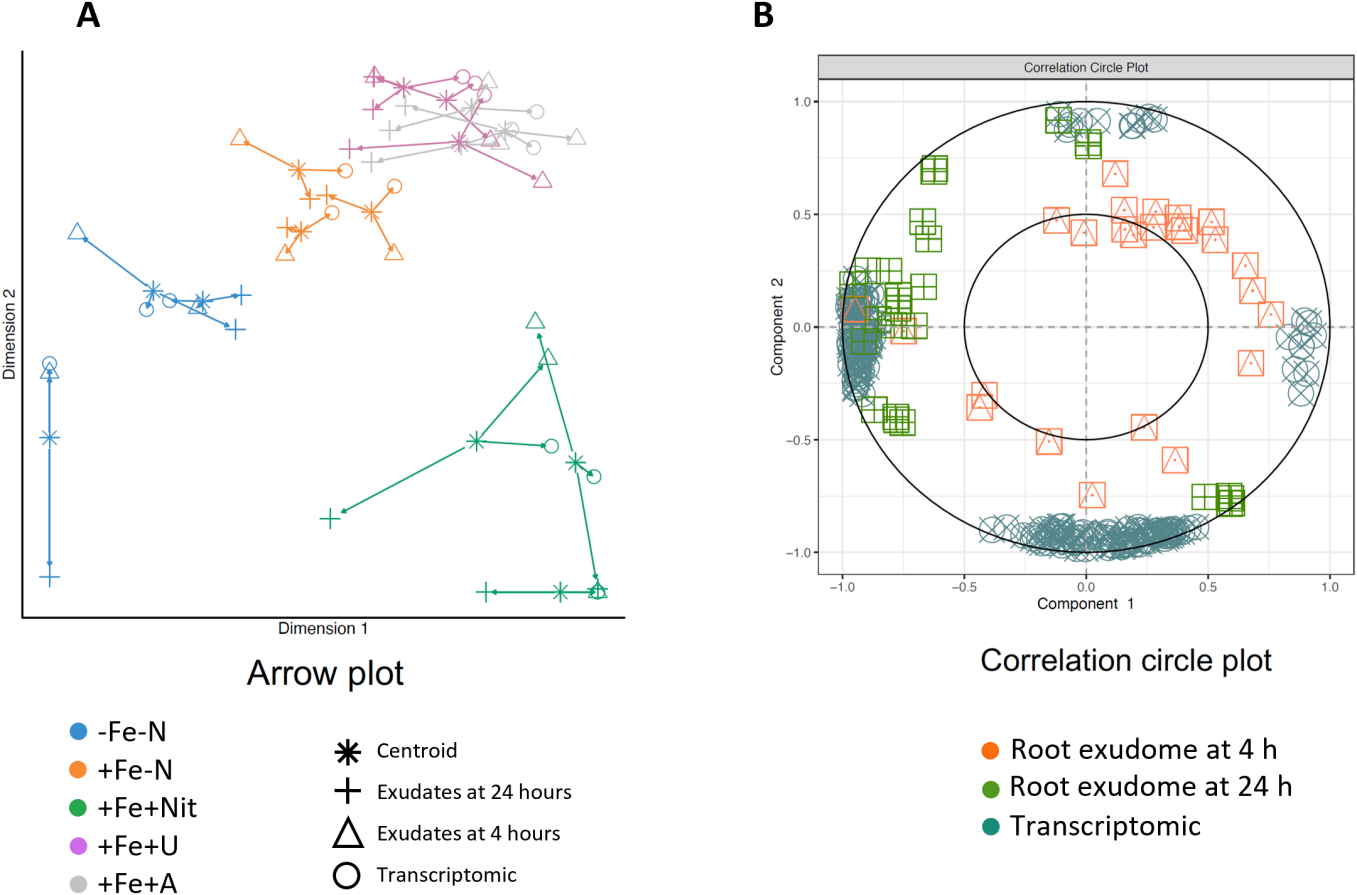
Multiomics data integration of root exudate datasets (4 and 24 h) and transcriptomic data. Arrow plot (A) of omics datasets in the first two components, and correlation circle plot (B) representing the correlation degree among omics datasets.

**FIGURE 7:**
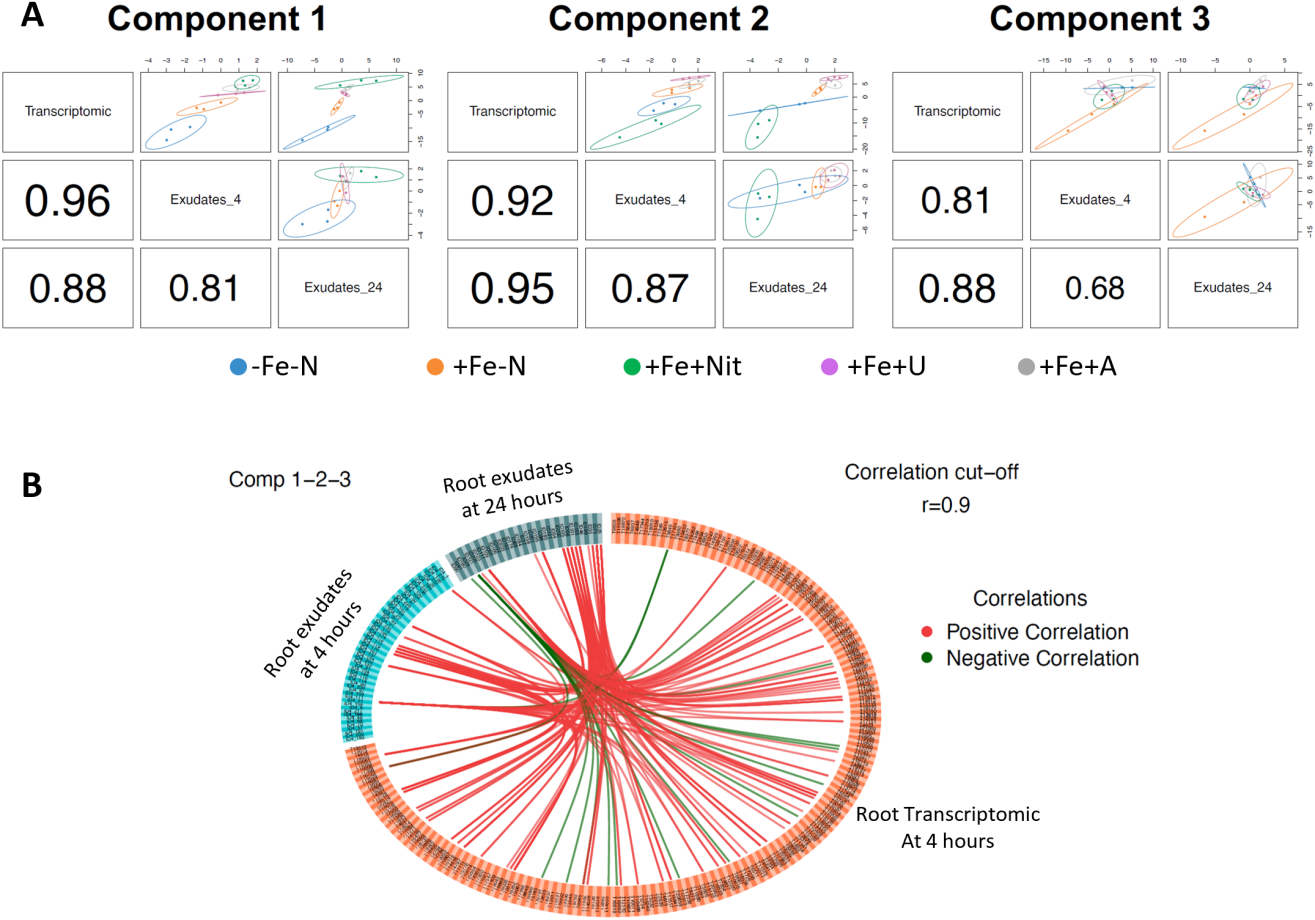
(A) Pearson's correlation score between the omics datasets the first three components and (B) the Circos plot representing both positive (red line) and negative (green line) connecting features from different omics datasets.

The arrow plot analyses revealed a good separation among the treatments, as both transcriptomic and root exudates were strongly separated depending on the treatment (Figure 6A). The correlation circle plot (Figure 6B) indicated that the transcriptomic data were separated on both principal Components 1 and 2: whereas the root exudates collected at 4 h of treatment were mainly separated along Component 1, showing the highest value of positive correlation with the transcriptomic data along this component; on the other hand, the root exudates collected at 24 h were mainly discriminated by Component 2, displaying along this component the highest value of correlation with the transcriptomic data (Figures 6B and 7). The correlation analyses indicate that in the main part, a positive correlation occurs between transcript and metabolites with very high score values on Components 1 and 2, as they ranged from 0.88 up to 0.96 (Figure 7). The clustering analysis of the 30 main discriminant compounds/transcripts of Component 1 highlighted a clear separation between the treatments, particularly showing a distinct separation of the −Fe–N from the other treatments (Figure S8A). Among the main discriminant compounds this analysis revealed the occurrence of some transcripts: encoding PAL, a transcription factor (BEL), mannan synthase (CSLA), protein kinases (CDPK, MAP3K‐RAF); metabolites (from 4 h root exudates): saponins (avenacin), flavonoids (Cyanidin 3‐O‐(6″‐caffeoyl‐glucoside)), tricarboxylic acid (avenic acid and deoxymugineic acid), terpenoid (brachialactone), lignan (Anhydro‐secoisolariciresinol); metabolites (from 24 h root exudates): fatty acid (palmitic acid), glucosinolate (6‐methylthiohexylglucosinolate), chalcones (Xanthohumol), flavones (Apigenin 7‐O‐glucuronide, Nobiletin/3‐Methoxysinensetin, Quercetin 4′‐O‐glucoside, Myricetin 3‐O‐rutinoside/Quercetin 3,4′‐O‐diglucoside/Quercetin 3‐O‐sophoroside), anthocyanidins (Delphinidin 3‐O‐(6″‐acetyl‐galactoside)/Delphinidin 3‐O‐(6″‐acetyl‐glucoside), Delphinidin 3‐O‐sambubioside, Cyanidin 3‐O‐(6″‐caffeoyl‐glucoside)/Delphinidin 3‐O‐(6″‐p‐coumaroyl‐glucoside)) (Table S3E).

The clustering of the 30 main discriminant compounds/transcripts of Component 2 indicated a clear separation of the +Fe + Nit from the other treatments (Figure S8B).

Whereas the 30 main discriminant compounds and transcripts of Component 3 suggested only a partial separation of the resupplied Fe treatment (+Fe–N) from the other nutritional conditions (Figure S8C).

**FIGURE 8:**
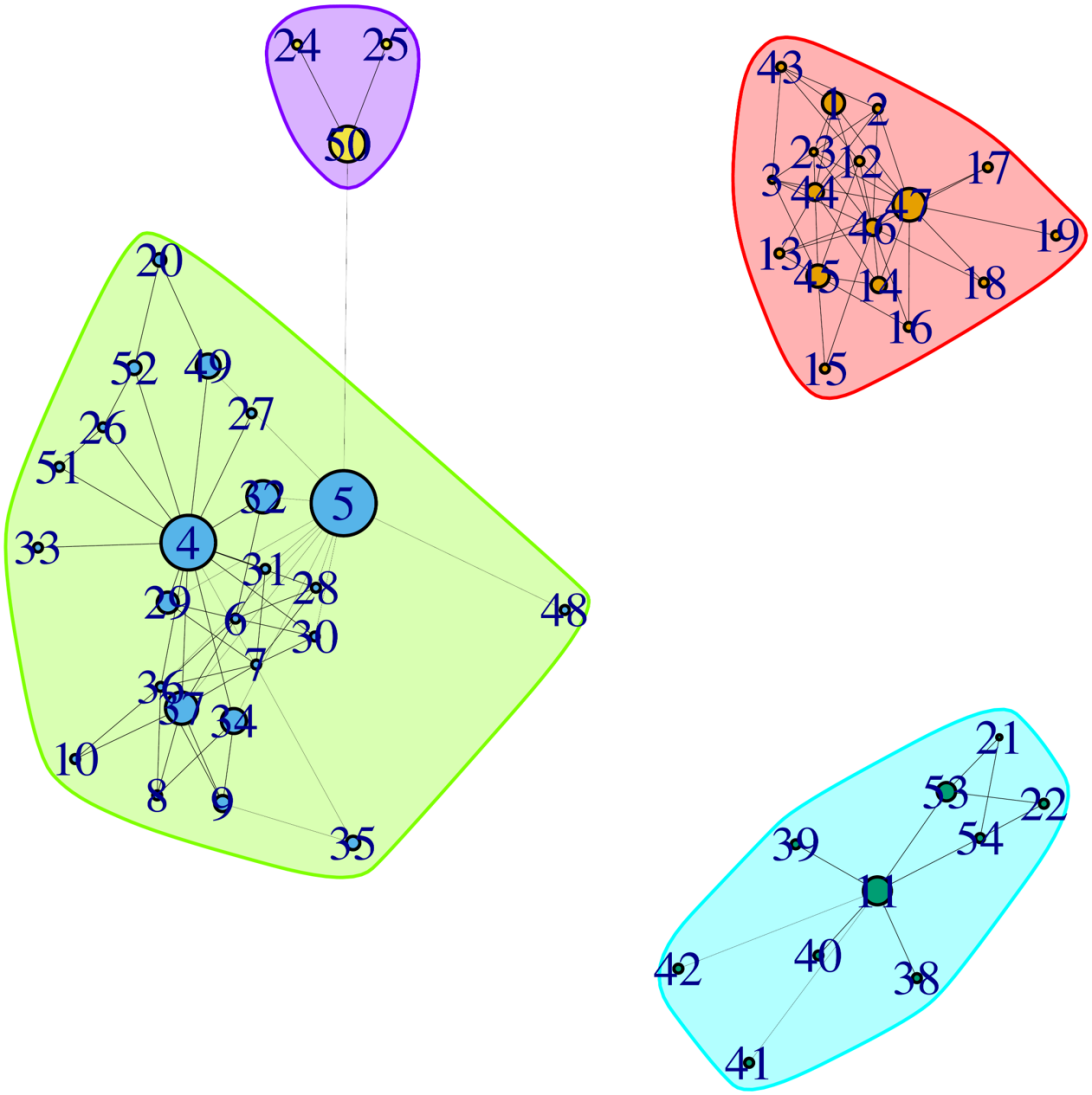
Integration of Omics datasets by DIABLO model: Network of the highly correlated features (betweenness centrality network with the community detection, key biomarkers; *r* > |0.9|). The sample node description is present as Table S6F. *Cluster purple:* (24_E4) 6″‐O‐Malonylgenistin; (25_E4) Jaceidin 4′‐O‐glucuronide; (50_E24) Quercetin 3‐O‐(6″‐malonyl‐glucoside) 7‐O‐glucoside. *Cluster green:* (4_T) hydrolase; (5_T) GAUT1:GAUT7 galacturonosyltransferase heterodimer; (6_T) Unknown; (7_T) transcription factor (BBR/BPC); (8_T) proteolytic phenylalanine ammonia‐lyase regulator (KFB‐PAL); (9_T) Unknown; (10_T) transcription factor (bZIP); (20_T) Unknown; (26_E4) Delphinidin 3‐O‐(6″‐malonyl‐glucoside); (27_E4) Vitisin A; (28_E4) Pelargonidin 3‐O‐rutinoside/Pelargonidin 3‐O‐sophoroside; (29_E4) Naringin/Narirutin; (30_E4) 3‐Hydroxyphloretin 2′‐O‐xylosyl‐glucoside; (31_E4) Cyanidin 3‐O‐(6″‐dioxalyl‐glucoside); (32_E4) Luteolin 7‐O‐rutinoside; (33_E4) Verbascoside; (34_E4) Petunidin 3‐O‐rutinoside; (35_E4) Delphinidin 3,5‐O‐diglucoside/Delphinidin 3‐O‐glucosyl‐glucoside; (36_E4) Quercetin 3‐O‐(6″‐malonyl‐glucoside) 7‐O‐glucoside; (37_E4)Kaempferol 3‐O‐(6″‐acetyl‐galactoside) 7‐O‐rhamnoside; (48_E24) Capsanthin; (49_E24) Eriocitrin/Neoeriocitrin; (51_E24) Kaempferol 3‐O‐(6″‐acetyl‐galactoside) 7‐O‐rhamnoside; (52_E24) Petunidin 3,5‐O‐diglucoside. *Cluster red*: (1_T) oxidoreductases; (2_T) Unknown; (3_T) Unknown; (12_T) Unknown; (13_T) Unknown; (14_T) C‐glucosyltransferase (CGT); (15_T) Unknown; (16_T) Unknown; (17_T) Unknown; (18_T) Unknown; (19_T) Unknown; (23_E4) GR24; (43_E24) Quercetin 4′‐O‐glucoside; (44_E24) Nobiletin/3‐Methoxysinensetin; (45_E24) Apigenin 7‐O‐glucuronide; (46_E24) 6‐methylthiohexylglucosinolate; (47_E24) Delphinidin 3‐O‐(6″‐acetyl‐galactoside). *Cluster cyan:* (11_T) Unknown; (21_T) transferase transferring sulfur‐containing group; (22_T) Unknown; (38_E24) Piceatannol; (39_E24) Carnosol/nomilactone B; (40_E24) brachialactone; (41_E24) Curcumin; (42_E24) 6‐methylthiohexyldesulfoglucosinolate; (53_E24) 1,2‐Disinapoylgentiobiose; (54_E24) Cyanidin 3‐O‐glucosyl‐rutinoside. T, refers to transcripts; E4, refers to root exudates collected at 4 h; E24, refers to root exudates at 24 h.

The network analysis (Figure 8) shows some interconnections between specific genes and exudated metabolites. In particular, the exudation of some flavonoids (flavonol: jaceidin and quercetin‐malonyl‐glucoside; and isoflavones: malonylgenistein) is linked to the expression of a transcript coding for a GAUT1:GAUT7 alpha 1,4‐galacturonosyltransferase heterodimer (glycosyltransferase). Data Integration also revealed a close connection among such phenolic compounds released after 4 h of treatment (anthocyanidins and flavonols: petunidin‐, delphinidin‐, quercetin‐, kaempferol‐glycosylated) and the expression of a gene coding for a proteolytic regulator of the phenylalanine ammonia‐lyase (KFB‐PAL, Solyc10g080610) and two transcription factors (a bZIP Solyc10g054010 and a BBR/BPC Solyc08g076230, Figure 8).

## 4 Discussion

In the present study, the tomato response to Fe resupply was evaluated under the applied N forms: ammonium, nitrate, or urea. In order to evaluate correlations between molecular and physiological datasets, variations in their root exudates and transcriptomic profiles were analyzed after 4 h of treatment. Moreover, the root exudates were also analyzed after 24 h to assess if the early molecular responses have outcomes in later time.

### 4.1 ZAT12‐Like Gene Is Modulated by Fe Resupply

The resupply of the micronutrient (Fe) to Fe‐deficient tomato plants induced after 4 h an upregulation of Strategy‐I Fe‐responsive genes (like *IRT1*, *IRT2*, *FRO*) independently of the N availability. This pattern agrees with previous observations showing that the availability of the metal transiently stimulates Fe‐deficient plants to overexpress the genes involved in its acquisition mechanisms. This induction is expected to be feedback‐regulated within 24 h of Fe resupply (Tomasi et al. 2013), although the timing can be dependent on the N form applied. After 24 h of Fe resupply, the presence of urea or ammonium no longer showed an upregulation of *IRT1*, whereas in the presence of nitrate, an upregulation of the expression of the gene was still observed in tomato roots. Regarding ferric chelate reductase activity, after 24 h, all three N forms showed lower activity compared to plants maintained under Fe‐deficiency conditions (Lodovici et al. 2024).

Lodovici et al. (2024) described that the application of urea, nitrate, or ammonium, along with Fe, induced in Fe‐deficient tomato plants (in both roots and leaves) an increased biosynthesis of phenylpropanoids (e.g., as observed by an increase of lignin and suberin precursors, flavonoids, and anthocyanin glycosides). Supporting this evidence, our results indicate that already after 4 h genes like *F6′H1* and *S8H* (involved in the biosynthesis of Fe‐mobilizing coumarins derived from the phenylpropanoid pathway) were upregulated and their expression is known to be crucial in root exudate biosynthesis. Phenol‐ and coumaric acid‐derivatives have been demonstrated to participate to the Fe‐acquisition strategy: thanks to their efflux, their presence in the rhizosphere allows the chelation and reduction of the metal in the rhizosphere (Schmid et al. 2014; Tsai and Schmidt 2017; Robe et al. 2025; in *Solanaceae* species *Nicotiana tabacum* ; Lefèvre et al. 2018).

Regarding the regulatory pathway, the Fe resupply (regardless of the N availability) modulated the expression of transcription factors, like *bHLH67* (upregulated in FRG region) and *ZAT12‐like* (downregulated). In tomato, *bHLH67* (together with bHLH66) has been reported to participate both in the interaction with FER and in its activation (Chen et al. 2022). ZAT12 in Arabidopsis is known to interact with FER, repressing the Fe‐deficient response (Le et al. 2016; Riaz and Guerinot 2021). A maize gene (*ZFP16‐1*, *GRMZM2G001205* and homologous to the Arabidopsis *ZAT12* gene) was found to be upregulated by N availability (Zanin et al. 2015). These results shed light on the role of ZAT12‐like transcription factors in the regulation of N and Fe responses.

### 4.2 UMAMIT Genes Are Modulated by Fe and N Supply

Transcriptomic results indicate that, when Fe is resupplied along with N, a subset of N‐responsive genes was modulated irrespective of the N‐form applied (urea, ammonium, or nitrate, called NRG). As expected, some genes related to N assimilation were found upregulated (as those coding for AMT, GS, GOGAT), whereas nitrate efflux transporter and other transporters were downregulated. The modulation induced by N supply appears evident by omics‐data integration as the Arrow plot of transcriptomic and metabolomic analyses shows a good separation among treatments (with urea‐ and ammonium‐treatments clustering together: +Fe + U and +Fe + A, Figure 6). This indicates that the modulation of the Fe‐deficiency response of tomato plants is dependent not only on Fe availability, but also on N availability and the N form applied in the nutrient solution (reduced‐N or oxidized‐N), at both levels: transcriptomic and metabolomic. For example, nitrate upregulated genes coding for a nitrate reductase and a carbonic anhydrase, whereas reductive‐N forms (as ammonium or urea) downregulated these genes. Vice versa, an *UMAMIT* transcript (*Solyc04g011340*) was upregulated by reductive‐N forms and downregulated by nitrate. Recently, some UMAMITs (Usually Multiple Amino acids Move In and out Transporter) have been characterized, which encode bidirectional amino acid transporters and tonoplast auxin transporters, some of them are expressed in roots (Fang et al. 2022). The transcript *Solyc04g011340* is homologous to *AtUmamiT14*, which protein is localized at the plasma membrane (Müller et al. 2015; Besnard et al. 2016), suggesting its involvement in the root amino acid secretion (Besnard et al. 2016, 2018). Recently, also *AtUmamiT30* has been demonstrated to be involved in the root release of amino acids (Agorsor et al. 2026). In this study, other UMAMIT transporters were transcriptionally modulated by either Fe supply or N forms (see Figure 4). *Solyc04g025750* (in FRG region, homologous to *AtUmamiT36*) was found to be upregulated by Fe deficiency, in agreement with Chen et al. (2022), and retroregulated by Fe resupply only when applied in combination with reductive N forms (urea or ammonium), whereas the addition of nitrate kept it upregulated. Our results, along with literature evidence (Zhao et al. 2021; Chen et al. 2022), suggest that some members of this transporter family are responsive to N and Fe availability in the external media, they influence the composition of the root exudome and might enhance the bioavailability of nutrients.

### 4.3 The Resupply of Fe and Urea Upregulated FER and SlbHLH66

The presence of urea in the nutrient solution induced the upregulation of the expression of some genes known to be involved in the N acquisition and specifically related to the urea metabolic and acquisition pathway (*TIP*, *PIP*, *Arginase*, *AAT*). Along with *Arginase*, the upregulation of the expression of genes coding for other downstream enzymes was identified (*polyamine oxidase*, *ODC* and *spermidine synthetase*), suggesting that urea acquisition by roots triggers the activation of polyamine synthesis. On the other hand, some genes related to the nitrate acquisition pathway were downregulated by urea, such as *NRTs*, *NAR2.1*, and *GLN2*. This pattern supports the idea of a cytosolic assimilation of ureic‐N based on a GS1‐AS pathway, which operates in parallel to the plastidic one for the assimilation of nitrate‐derived ammonium.

Among those transcripts specifically modulated by urea and Fe supply, the transcription factor FER was found upregulated (*Solyc06g051550*, in agreement with Lodovici et al. 2024) along with *SlbHLH66* (*Solyc10g079650*). This latter has been reported to be interacting with other bHLHs (*bHLH67*, *Solyc10g079660*, upregulated in FRG region) as well as activating FER (Chen et al. 2022). Moreover, that response is enhanced by the downregulation of the expression of *ZAT12‐like*, as previously described. This might explain that the urea treatment leads to the highest *LeIRT2* expression level (*Solyc02g069190*), which is a known target of FER induction (Mai et al. 2016; Wu and Ling 2019; Figure 4).

Data indicate the modulation of the S assimilation pathway by urea, as an upregulation of the expression of *APR* and a concomitant downregulation of *APK* was observed. This evidence suggests a metabolic shift in favor of cysteine synthesis at the expense of sulfur‐containing secondary metabolites. Nevertheless, S‐adenosyl methionine synthetase, a key enzyme in the Yang cycle, was found to be downregulated by urea treatment. This behavior might have determined a reduction of NA synthesis and therefore a very low capability to translocate Fe, resulting in the lowest Fe accumulation in leaves of urea treated plants in comparison to ammonium or nitrate treated ones (Lodovici et al. 2024).

Several transcripts related to the cell wall biosynthesis were found modulated (in particular four pectin methylesterase, and two xylan O‐acetyltransferase (XOAT) were upregulated). Factors that influence the synthesis of root cell walls are likely to affect Fe storage in the apoplast and its later reuse by plants (Ye et al. 2015). The increased retention of Fe in the root apoplast during Fe deficiency appears to be linked to a reduced level of pectin methyl‐esterification. In contrast, the Fe supply to Fe‐deficient plants can determine the opposite effect, increasing the positive charges on the apoplastic space in order to limit the Fe retention and promote its acquisition. This aspect could deserve more attention in relation to the pH changes induced by the N form applied in the apoplastic medium. Nitrate acquisition causes the alkalinization of the apoplastic space, whereas ammonium nutrition induces its acidification; urea supply has a mild effect on apoplastic pH (Buoso et al. 2021). Thus, pH effects and changes in cell wall composition may explain why the highest levels of Fe accumulation were found in roots after 24 h of Fe supply with nitrate, then with urea, and the lowest with ammonium (Lodovici et al. 2024).

### 4.4 Ammonium Triggers the Overexpression of Genes Coding for Amino Acid and Flavonoid Transporters

In agreement with literature (Britto and Kronzucker 2005), two *PEP carboxylase* genes were upregulated by ammonium and one of them was also found to be responsive to Fe‐deficiency (Solyc10g007290.4.1, Chen et al. 2022). The activity of the encoded enzymes is related to the regulation of root cytosolic pH.

The supply of Fe along with ammonium induced the specific modulation of the expression of genes linked to Fe‐deficiency response (Chen et al. 2022), such as the upregulation of *VIT*, *FFK*, *PEPC*. Besides, even transcripts related to other nutritional pathways, such as those of P and S, were modulated, such as glutathione‐S‐transferase (also found modulated by Fe starvation response in tomato, Chen et al. 2022), S‐adenosyl methionine decarboxylase, and SULTRs (A‐NRG region, Figure 4).

Among transporters linked to root exudation, the occurrence of ammonium induced the upregulation of transcripts coding for MATEs and UMAMITs. MATE homologous in Arabidopsis and lupin have been characterized to be involved in the root release of organic acids (citrate, FRD3 Roschzttardtz et al. 2011) and phenol‐derivatives (flavonoids, Biała‐Leonhard et al. 2021). Two transcripts (*Solyc10g080980*, *Solyc11g012930*) coding for UMAMIT transporters were specifically induced by ammonium treatment. Both these transcripts were previously described to be Fe‐deficiency responsive in tomato (Chen et al. 2022), thus, this upregulation by ammonium treatment might increase the effect of these UMAMIT transporters.

### 4.5 Nitrate Activates Its Acquisition Pathway and Downregulates the Acquisition of Reductive N Forms

The supply of Fe along with nitrate upregulated genes involved in the N‐acquisition pathway, such as *AHA1*, *NPF4.4*, *NiR*, *GDH*, *AAT*. On the other hand, urea and ammonium transporters were downregulated by nitrate (*DUR3* and *AMT1;1*, in agreement with Zanin et al. 2015; Hao et al. 2020 in maize).

The supply of nitrate and Fe specifically induced two *UMAMIT* genes (one transcript was up‐ and the other downregulated by nitrate). As shown by Chen et al. (2021), the first one (*Solyc03g080070*) is known to be responsive to Fe availability.

The omics‐data integration indicated that the modulation induced by Fe and nitrate‐containing treatment characterized the Component 2 (Figure S6). Among the most representative transcripts, those encoding flavonol synthase/flavanone 3‐hydroxylase, glutathione S‐transferase, PP1 phosphatase, nitrate reductase, solute transporter (UMAMIT), carbonic anhydrase, nitrate transporter (NRT2), and E3 ubiquitin‐protein ligase ATL23 were identified. Relating to the root exudates at both 4 and 24 h, some phenolic compounds, terpenes, and organic acids were identified (in particular, after 24 h, gluconic acid, curcumin, and quercetin; these compounds are known to display a metal chelating activity). It is worth highlighting the differential expression of genes encoding enzymes and transporters involved in both root exudation and N assimilation upon Fe resupply in the presence of nitrate, suggesting a coordinated regulation between these processes.

### 4.6 Description of the Exudomic Profile and Its Correlation With the Transcriptome

The integration of omics datasets (transcriptomic and exudomic) indicates that Fe resupply had a pronounced impact on the transcriptomic and exudomic profiles in tomato roots. Indeed, the −Fe–N condition was mainly characterizing the Component 1 (Figure S6). After 4 h the biomarkers that mainly characterized the root exudome were DMA and avenic acid (two amino acid derivatives possessing Fe chelating activities, Fushiya et al. 1980; Table S3), along with brachialactone (an inhibitor of biological nitrification) and avenacin (an antifungal saponin derivative) that can have a role as allelochemicals to preserve the nutrient availability for tomato plants. In *Strategy I* plants (including tomato plants), the occurrence of DMA in the root exudates was previously observed in tomato and in grapevine (Marastoni et al. 2020; Astolfi et al. 2020), however, their biosynthesis and release from dicots still need further investigations.

The Component 1 (Figure S6) was mainly defined by the following transcripts coding for PAL, protein phosphorylase, transcription factor (BEL), and oxidoreductase, underlying the important role of phenylpropanoid derivatives and their regulation and signaling pathways for the plant's response to Fe deficiency. The gene modulation characterizing Component 1 is also reflected in the long term, as the root exudate after 24 h was predominantly characterized by the presence of phenolic derivatives, some of which are known to have Fe‐reducing and chelating properties (quercetin, cyanidin, apigenin).

The multiomics approach allowed us to generate a DIABLO model (Figure 8) indicating the involvement of such genes (of known and unknown functions) that might play a key role in defining plant response to N and Fe supply. The exudation of flavonoids (flavonol: jaceidin and quercetin‐malonyl‐glucoside; isoflavones: malonylgenistein) is linked to the expression of a transcript coding for a GAUT1:GAUT7 alpha 1,4‐galacturonosyltransferase heterodimer (glycosyltransferase). This enzyme is related to the biosynthesis of galacturonans, which are constituents of the cell wall. In Arabidopsis, Peng et al. (2021) demonstrated that rhamnogalacturonan‐II (RG‐II) galactosylation is required for the apoplastic Fe reallocation. In particular, the gene *AtCdi (*coding for a glycosyltransferase required for the transfer of GDP‐l‐galactose to the terminus of side chain A on RG‐II) was found to be expressed in roots and upregulated under Fe deficiency. In the absence of this enzyme, the authors observed a reduction of RG‐II dimerization and the disruption of cell wall formation, and the incapacity to reallocate apoplastic Fe from roots to Arabidopsis shoots. In the apoplast, the Fe deficiency‐induced phenolic compounds secretion plays a key role in the reutilization of the root apoplastic Fe, for the metal chelation and reduction (Jin et al. 2007). Hence, the coordination between the two processes (the root release of flavonoids along with the modifications of the cell wall structure) might play a crucial role for an efficient usage of the cell wall Fe pool. Moreover, the network analysis also revealed a close connection among mainly phenolic compounds released at 4 h and the expression of proteolytic regulator of the PAL (KFB‐PAL Solyc10g080610), and two transcription factors (bZIP Solyc10g054010 and BBR/BPC Solyc08g076230, Figure 8).

In the root exudome, several phenolic derivatives have been identified, and their synthesis depends on the phenylpropanoid pathway. The latter is posttranscriptionally regulated, such as the ubiquitination and proteasome‐mediated degradation of PAL proteins activated by Kelch Domain F‐Box (KFB) proteins (Dong and Lin 2021). In Arabidopsis, the involvement of a KFB as a negative regulator of flavonoid biosynthesis has been reported (Kim et al. 2020). The identification of a KFB protein along with some phenolic derivatives in the root exudome at both 4 and 24 h indicates that their biosynthesis and release are involved in the plant response to Fe and N supply. The transcriptomic profiles indicate the metabolic activation of the coumarin branch of the phenylpropanoid pathway as two *F6′H1* transcripts and *S8H* were upregulated by Fe supply. These enzymes are involved in the biosynthesis of the coumarins, like fraxetin and sideretin, known to participate in Fe mobilization processes in the rhizosphere of *Strategy I* plants (Zouari et al. 2001; Vélez‐Bermúdez and Schmidt 2023). Our results suggest that the form of N supplied along with Fe may influence the activation of this pathway as nitrate and urea treatments induced higher expression levels of *F6′H1* transcripts compared with ammonium, potentially promoting coumarin biosynthesis and their release into the rhizosphere.

One transcription factor (*Solyc10g054010* coding a bZIP, Figure 8) is homologous to the Arabidopsis basic leucine‐zipper 44, which has been recently identified to cooperate with MYB transcription factors to modulate the plant response to Fe stress (Wu et al. 2024). bZIP44 increases the abilities of MYB10 and MYB72 to bind NAS promoters, and hence it stimulates the nicotianamine biosynthesis. In Lodovici et al. (2024) nicotianamine was highly present as internal metabolites in tomato plants, even though not significantly modulated by N form and Fe treatments.

For the best of our knowledge the involvement of Barley B Recombinant/Basic PentaCysteine (BBR/BPC) transcription factors (Solyc08g076230) in the Fe‐deficiency response in plants has not yet been investigated. BBR/BPC transcription factor family in plants binds to “GA” repeats, akin to animal GAGA Factors (GAFs). These GAGA‐binding proteins are some of the rare transcription factors that control genes at various stages by altering chromatin structure (Sahu et al. 2023). Recent findings in plants (Arabidopsis and rice) have highlighted the occurrence of a chromatin‐based regulation of Fe homeostasis in plants (reviewed by Su et al. 2022).

Network analysis indicates that the metabolites released at 24 h are mainly connected with unknown transcripts (cluster red and cyan in Figure 8). Investigating these transcripts and their potential role in root exudation could provide valuable insight in future studies.

## 5 Conclusions

This study demonstrated that the root response of tomato plants to Fe resupply is strongly influenced by the applied N form. The integration of transcriptomic and metabolomic data of root exudates reveals coordinated changes in the regulatory networks and metabolic pathways associated with Fe acquisition. In particular, the modulation of transcription factors (such as bZIP, MYB, and BEL), transport processes (UMAMITs, MATEs), and phenylpropanoid‐derived metabolites highlight the activation of mechanisms involved in root exudation and nutrient acquisition. Overall, this work provides new insight into the interaction between N nutrition and Fe acquisition mechanisms in tomato.

In the context of sustainable agriculture, these findings highlight the importance of N forms in the modulation of the root metabolic responses and rhizosphere processes that contribute to nutrient acquisition and plant resilience under conditions of limited micronutrient availability.

## Author Contributions

All authors contributed to the study conception, design, data collection, analyses, and manuscript preparation. All authors read and approved the final manuscript.

## Funding

This work was supported by Università degli Studi di Udine, Centre Mondial de l’Innovation Roullier, and European Union Next‐GenerationEU (PNRR_M4C2Inv1.4_CN00000022).

## Conflicts of Interest

The authors declare no conflicts of interest.

## Supporting information


**Table S1:** List of transcripts for each Venn diagram region referred to the RNAseq analyses of tomato roots after 4 h of treatment.
**Table S2:** KEGG enrichment analyses of modulated transcripts (up‐ or downregulated transcripts) in +Fe–N, +Fe + A, +Fe + Nit, +Fe + U in comparison to −Fe–N.
**Table S3:** Enrichment GO analyses of modulated transcripts (total, up or down modulated transcripts) in +Fe + U, +Fe + A, +Fe + Nit in comparison to −Fe–N, and +Fe + U, +Fe + A, +Fe + Nit in comparison to +Fe–N.
**Table S4:** Dataset exudates collected after 4 h of Fe and N resupply (A); dataset exudates collected after 24 h of Fe and N resupply (B); VIP makers selected from OPLS‐DA model for exudates collected after 4 h of Fe and N resupply (C); VIP makers selected from OPLS‐DA model for exudates collected after 24 h of Fe and N resupply (D); data integration results, reporting features highly correlated: transcriptomic at 4 h, root exudomic at 4 h and root exudomic at 24 h (E); and sample ID node description of the network analysis (F).
**Table S5:** Root exudates by tomato plants after 4 h from the supply of Fe and N forms.
**Table S6:** Root exudates by tomato plants after 24 h from the supply of Fe and N forms.
**Table S7:** List of the 15 most downregulated DEGs for each comparison.
**Table S8:** List of the 15 most upregulated DEGs for each comparison.
**Figure S1:** The root transcriptomic profiles are visualized as heatmap with dendrogram showing clustering of samples (data expressed as Log_10_FPKM, A). The volcano plots indicated the distribution of significant DEGs (Log_2_|FC| ≥ 1.00, *N* = 3, *q* ≤ 0.05) in the four comparisons: +Fe–N vs. −Fe–N (B); +Fe + Nit vs. −Fe–N (C); +Fe + A vs. −Fe–N (D); +Fe + U vs. −Fe–N (E).
**Figure S2:** GO enrichment analyses of modulated transcripts performed on AgriGO of the following comparisons: +Fe + Nit, +Fe + U, +Fe + A vs. −Fe–N.
**Figure S3:** GO enrichment analyses of modulated transcripts performed on AgriGO of the following comparisons: +Fe + Nit, +Fe + U, +Fe + A vs. + Fe–N.
**Figure S4:** Hierarchical cluster analysis of metabolites in the root exudates collected after 4 h (A) or after 24 h (B).
**Figure S5:** Organic acid concentration in root exudates of tomato plants after 4 and 24 h of treatment (Holm–Siddak method, one way‐ANOVA, *N* = 3, *p* < 0.05).
**Figure S6:** Heatmaps of Component 1 (A), Component 2 (B), and Component 3 (C) and bar plots of the contribution of the max. 10 most characterizing “features” (modulated transcript, or metabolic exudate at 4 h or metabolic exudate at 24 h) for each component.
**Text S1:** Additional details about materials and methods.

## Data Availability

All data that support the findings of this study are included in this published article or openly available at the Gene Expression Omnibus (https://www.ncbi.nlm.nih.gov/geo/), under the accession GSE294740.
