# Supplementary material for: The Early Response to Urea, Nitrate, or Ammonium and Iron Resupply in Tomato Roots Highlights the Induction of FER, bHLHs, UMAMITs, and MATEs
Source: Physiol Plant. 2026 Jul 22;178(4):e70977. doi: 10.1111/ppl.70977 (PMC13390592; doi:10.1111/ppl.70977)
Supplement: Supplementary file 1 — Table S1: List of transcripts for each Venn diagram region referred to the RNAseq analyses of tomato roots after 4 h of treatment. Table S2: KEGG enrichment analyses of modulated transcripts (up‐ or downregulated transcripts) in +Fe–N, +Fe + A, +Fe + Nit, +Fe + U in comparison to −Fe–N. Table S3: Enrichment GO analyses of modulated transcripts (total, up or down modulated transcripts) in +Fe + U, +Fe + A, +Fe + Nit in comparison to −Fe–N, and +Fe + U, +Fe + A, +Fe + Nit in comparison to +Fe–N. Table S4: Dataset exudates collected after 4 h of Fe and N resupply (A); dataset exudates collected after 24 h of Fe and N resupply (B); VIP makers selected from OPLS‐DA model for exudates collected after 4 h of Fe and N resupply (C); VIP makers selected from OPLS‐DA model for exudates collected after 24 h of Fe and N resupply (D); data integration results, reporting features highly correlated: transcriptomic at 4 h, root exudomic at 4 h and root exudomic at 24 h (E); and sample ID node description of the network analysis (F). Table S5: Root exudates by tomato plants after 4 h from the supply of Fe and N forms. Table S6: Root exudates by tomato plants after 24 h from the supply of Fe and N forms. Table S7: List of the 15 most downregulated DEGs for each comparison. Table S8: List of the 15 most upregulated DEGs for each comparison. Figure S1: The root transcriptomic profiles are visualized as heatmap with dendrogram showing clustering of samples (data expressed as Log10FPKM, A). The volcano plots indicated the distribution of significant DEGs (Log2|FC| ≥ 1.00, N = 3, q ≤ 0.05) in the four comparisons: +Fe–N vs. −Fe–N (B); +Fe + Nit vs. −Fe–N (C); +Fe + A vs. −Fe–N (D); +Fe + U vs. −Fe–N (E). Figure S2: GO enrichment analyses of modulated transcripts performed on AgriGO of the following comparisons: +Fe + Nit, +Fe + U, +Fe + A vs. −Fe–N. Figure S3: GO enrichment analyses of modulated transcripts performed on AgriGO of the following comparisons: +Fe + Nit, +Fe + U, +Fe + A vs. + [file PPL-178-e70977-s001.docx]

**The Early Response to Urea, Nitrate, or Ammonium and Iron Resupply in Tomato Roots Highlights the Induction of FER, bHLHs, UMAMITs and MATEs**

Arianna Lodovici¹†; Leilei Zhang²†; Nicola Tomasi¹; Gabriella Vinci¹; Fabio Marroni¹; Barbara Piani¹; Fatemeh Hassanvand¹; Mustapha Arkoun³; Luigi Lucini²; Laura Zanin¹*

^†^ equally contributed

**SUPPLEMENTARY MATERIAL**

**Supplementary Text S1.**

***Root exudation analyses***

The root exudates were profiled through a 6560-drift tube-ion mobility-quadrupole-time of flight-high resolution mass spectrometer (DTIM-UHPLC-QTOF-HRMS; Agilent Technologies, Santa Clara, CA, USA). The chromatographic separation was achieved under a water-acetonitrile (both LC-MS grade, from Sigma-Aldrich, Milan, Italy) gradient elution (6–94 % acetonitrile in 32 min), flow rate of 0.2 mL/min and injection volume of 6 μL, using 0.1 % (v/v) formic acid as phase modifier on an -Agilent Poroshell 120 PFP column (100 mm × 2.1 i.d., 1.9 μm particle size) -Agilent Zorbax Eclipse plus C18 analytical column (50 × 2.1 mm, 1.8 μm).

The QTOF mass analyzer operated in positive mode (ESI+) for both MS and MS/MS acquisition with nitrogen as both sheath gas (12 L/min and 315 ◦C) and drying gas (14 L/min and 250 ◦C). The nebulizer pressure was 45 psi, nozzle voltage was 350 V, and the capillary voltage was 4.0 kV. For MS acquisition, the full scan mode was performed within range of the m/z 100–1200 (1 spectra/s), mass resolution of 30,000 full width at half maximum (FWHM), m/z = 200. The Processing of the chromatograms was carried out using the MassHunter Qualitative Analysis software (version B.06.00, Agilent Technologies).

The raw mass features were processed using Profinder B.07 (Agilent Technologies) software based on the “find-by-formula” algorithm. In this regard, compound annotation was recursively achieved, following mass and retention time alignment, against a homemade database based on literature information. The annotation was based on the isotopic profile of each molecular feature detected (consisting of a monoisotopic mass, isotope spacing, and ratio combination, with a mass accuracy of 5 ppm). Data filtering was used to remove the features not detected in at least 75 % of replications per each tested group. According to the Metabolomics Standards Initiative (MSI), confidence Level 2 of identification (i.e., putatively annotated compounds, COSMOS standards in metabolomics).

***Multivariate statistical analysis and chemometrics***

Mass Profiler Professional 15.1 software (Agilent Technologies) was used for data filtration (area threshold >10,000 counts), Log2-transformation, and normalization at the 75th percentile. The detected features were baselined to their median in the dataset. Unsupervised hierarchical cluster analysis (HCA) was conducted using a fold-change heat map (Euclidean similarity measure and ‘Wards’ as linkage rule). Normalized resulting data were Pareto-scaled for multivariate data analysis by SIMCA® version 17 software (Sartorius, Umeå, Sweden). The supervised orthogonal partial least squares discriminant analysis (OPLS-DA) was carried out. separating the variability between groups into predictive and orthogonal (i.e., ascribable to technical and biological variation). The confidence ellipses were built considering 95% and 99% limits. The goodness-of-fit (R2Y) and the goodness-of-prediction (Q2Y) parameters were considered to evaluate the quality of the models, setting a Q2 predictive ability > 0.5 as a threshold for good predictability. The multivariate models were validated using cross validation-analysis of variance (CV-ANOVA p-value < 0.05 for model significance), the permutation tests (200 random permutations) was performed to discard over- fitting. The variable importance in projection (VIP) selection method was used to select those compounds having the highest discrimination potential (VIP score > 1.00), which were putatively identified as markers. Fold change (FC) analyses, expressed as Log2(FC) values, were performed using the Mass Profiler Professional 15.1 software with a cut-off value of FC > 1.00.

**Supplementary Tables**

**Supplementary Table S1 (.xlsx file)**. List of transcripts for each Venn diagram region referred to the RNAseq analyses of tomato roots after 4 hours of treatment. The transcriptomic profiles of +Fe-N, +Fe+A, +Fe+U, +Fe+Nit were compared with the profile of -Fe-N (|Log_2_FC| ≥ 1.00, N = 3, q-value ≤0.05; in the first sheet is reported a picture of Venn diagram with the indication of the region number).

**Supplementary Table S2.** KEGG enrichment analyses of modulated transcripts (up- or down-regulated transcripts) in +Fe-N, +Fe+A, +Fe+Nit, +Fe+U in comparison to -Fe-N.

| **+Fe-N vs -Fe-N** | | | | |
| --- | --- | --- | --- | --- |
| **KEGG enrichment of UP-regulated DEGs** | | | | |
| *Pathways* | *Fold Enrichment* | *Enrichment FDR* | *nGenes* | *Pathway Genes* |
| ABC transporters | 38.9 | 0.00 | 4 | 23 |
| Cutin suberine and wax biosynthesis | 41.9 | 0.00 | 3 | 16 |
| Biosynthesis of various plant secondary metabolites | 41.9 | 0.00 | 3 | 16 |
| Biosynthesis of secondary metabolites | 3 | 0.01 | 10 | 733 |
|  |  |  |  |  |
| **KEGG enrichment of DOWN-regulated DEGs** | | | | |
| *Pathways* | *Fold Enrichment* | *Enrichment FDR* | *nGenes* | *Pathway Genes* |
| Phenylpropanoid biosynthesis | 6.3 | 0.04 | 4 | 95 |
| Metabolic pathways | 2.2 | 0.04 | 18 | 1238 |
| Biosynthesis of secondary metabolites | 2.5 | 0.04 | 12 | 733 |
| Plant hormone signal transduction | 5.4 | 0.04 | 5 | 139 |
| Carotenoid biosynthesis | 17.6 | 0.05 | 2 | 17 |

| **+Fe+A vs -Fe-N** | | | | |
| --- | --- | --- | --- | --- |
| **KEGG enrichment of UP-regulated DEGs** | | | | |
| *Pathways* | *Fold Enrichment* | *Enrichment FDR* | *nGenes* | *Pathway Genes* |
| Metabolic pathways | 2.9 | 0.00 | 26 | 1238 |
| Biosynthesis of secondary metabolites | 3.6 | 0.00 | 19 | 733 |
| Cysteine and methionine metabolism | 10.2 | 0.00 | 5 | 68 |
| Glycolysis/Gluconeogenesis | 8.5 | 0.00 | 5 | 82 |
| Biosynthesis of amino acids | 5.6 | 0.02 | 5 | 124 |
| Carbon fixation in photosynthetic organisms | 11 | 0.02 | 3 | 38 |
| Fructose and mannose metabolism | 9.3 | 0.03 | 3 | 45 |
| Carbon metabolism | 4.7 | 0.03 | 5 | 149 |
| Diterpenoid biosynthesis | 17.4 | 0.03 | 2 | 16 |
| Biosynthesis of various plant secondary metabolites | 17.4 | 0.03 | 2 | 16 |
| Sulfur metabolism | 15.5 | 0.03 | 2 | 18 |
| Starch and sucrose metabolism | 6.6 | 0.04 | 3 | 63 |
|  |  |  |  |  |
| **KEGG enrichment of DOWN-regulated DEGs** | | | | |
| *Pathways* | *Fold Enrichment* | *Enrichment FDR* | *nGenes* | *Pathway Genes* |
| Metabolic pathways | 2.9 | 0.00 | 53 | 1238 |
| Phenylpropanoid biosynthesis | 9.1 | 0.00 | 13 | 95 |
| Biosynthesis of secondary metabolites | 2.8 | 0.00 | 31 | 733 |
| Flavonoid biosynthesis | 7.9 | 0.02 | 4 | 34 |

| **+Fe+Nit vs -Fe-N** | | | | |
| --- | --- | --- | --- | --- |
| **KEGG enrichment of UP-regulated DEGs** | | | | |
| *Pathways* | *Fold Enrichment* | *Enrichment FDR* | *nGenes* | *Pathway Genes* |
| Metabolic pathways | 2.8 | 0.00 | 23 | 1238 |
| Biosynthesis of secondary metabolites | 3 | 0.00 | 15 | 733 |
| Glutathione metabolism | 13 | 0.00 | 4 | 46 |
| Lysine biosynthesis | 49.7 | 0.01 | 2 | 6 |
| Pentose phosphate pathway | 15.4 | 0.01 | 3 | 29 |
| Nitrogen metabolism | 21.3 | 0.03 | 2 | 14 |
| Biosynthesis of various plant secondary metabolites | 18.6 | 0.04 | 2 | 16 |
|  |  |  |  |  |
| **KEGG enrichment of DOWN-regulated DEGs** | | | | |
| *Pathways* | *Fold Enrichment* | *Enrichment FDR* | *nGenes* | *Pathway Genes* |
| Metabolic pathways | 2.5 | 0.00 | 30 | 1238 |
| Biosynthesis of secondary metabolites | 3 | 0.00 | 22 | 733 |
| Tropane piperidine and pyridine alkaloid biosynthesis | 13.2 | 0.02 | 3 | 23 |
| Plant-pathogen interaction | 5.2 | 0.02 | 6 | 117 |
| Phenylalanine metabolism | 10.9 | 0.02 | 3 | 28 |
| Phenylpropanoid biosynthesis | 5.3 | 0.02 | 5 | 95 |
| Biosynthesis of nucleotide sugars | 7 | 0.02 | 4 | 58 |
| Cysteine and methionine metabolism | 6 | 0.03 | 4 | 68 |
| Amino sugar and nucleotide sugar metabolism | 4.8 | 0.05 | 4 | 84 |

| **+Fe+U vs -Fe-N** | | | | |
| --- | --- | --- | --- | --- |
| **KEGG enrichment of UP-regulated DEGs** | | | | |
| *Pathways* | *Fold Enrichment* | *Enrichment FDR* | *nGenes* | *Pathway Genes* |
| Biosynthesis of secondary metabolites | 3.4 | 0.00 | 31 | 733 |
| MAPK signaling pathway-plant | 9.3 | 0.00 | 11 | 94 |
| Metabolic pathways | 2.5 | 0.00 | 39 | 1238 |
| Glycolysis/Gluconeogenesis | 6.8 | 0.00 | 7 | 82 |
| Plant hormone signal transduction | 5.1 | 0.00 | 9 | 139 |
| Arginine and proline metabolism | 8.6 | 0.01 | 4 | 37 |
| Biosynthesis of amino acids | 4.5 | 0.01 | 7 | 124 |
| Sulfur metabolism | 13.2 | 0.01 | 3 | 18 |
| Cysteine and methionine metabolism | 5.8 | 0.01 | 5 | 68 |
| Protein processing in endoplasmic reticulum | 4.5 | 0.01 | 6 | 106 |
| Alanine aspartate and glutamate metabolism | 7.9 | 0.04 | 3 | 30 |
| Biosynthesis of unsaturated fatty acids | 14.4 | 0.04 | 2 | 11 |
|  |  |  |  |  |
| **KEGG enrichment of DOWN-regulated DEGs** | | | | |
| *Pathways* | *Fold Enrichment* | *Enrichment FDR* | *nGenes* | *Pathway Genes* |
| Metabolic pathways | 3.3 | 0.0000 | 77 | 1238 |
| Biosynthesis of secondary metabolites | 3 | 0.0000 | 42 | 733 |
| Phenylpropanoid biosynthesis | 6.7 | 0.0000 | 12 | 95 |
| Ascorbate and aldarate metabolism | 8.2 | 0.0015 | 6 | 39 |
| Biosynthesis of nucleotide sugars | 6.4 | 0.0015 | 7 | 58 |
| Amino sugar and nucleotide sugar metabolism | 5.1 | 0.0022 | 8 | 84 |
| Beta-Alanine metabolism | 8.6 | 0.0027 | 5 | 31 |
| Flavonoid biosynthesis | 7.8 | 0.0037 | 5 | 34 |
| Tropane piperidine and pyridine alkaloid biosynthesis | 9.2 | 0.0065 | 4 | 23 |
| Phenylalanine metabolism | 7.6 | 0.0120 | 4 | 28 |
| Nitrogen metabolism | 11.4 | 0.0120 | 3 | 14 |
| Isoquinoline alkaloid biosynthesis | 11.4 | 0.0120 | 3 | 14 |
| Butanoate metabolism | 8.4 | 0.0260 | 3 | 19 |
| Phosphatidylinositol signaling system | 5.7 | 0.0260 | 4 | 37 |
| Biosynthesis of cofactors | 2.9 | 0.0300 | 8 | 145 |

**Supplementary Table S3 (.xlsx file)**. Enrichment GO analyses of modulated transcripts (total, up or down modulated transcripts) in +Fe+U, +Fe+A, +Fe+Nit in comparison to -Fe-N, and +Fe+U, +Fe+A, +Fe+Nit in comparison to +Fe-N.

**Supplementary Table S4 (.xlsx file)**. Dataset exudates collected after 4h of Fe and N resupply (**A**); Dataset exudates collected after 24h of Fe and N resupply (**B**); VIP makers selected from OPLS-DA model for exudates collected after 4h of Fe and N resupply (**C**); VIP makers selected from OPLS-DA model for exudates collected after 24h of Fe and N resupply (**D**); Data integration results, reporting features highly correlated: transcriptomic at 4 hours, root exudomic at 4 hours and root exudomic at 24 hours (**E**); Sample ID node description of the network analysis (**F**).

**Supplementary Table S5. Root exudates by tomato plants after 4 hours from the supply of Fe and N forms.** Value are expressed as Log_2_FC in comparison to -Fe-N treatment. The root exudome of +Fe/+Fe-N, +Fe+A, +Fe+Nit, +Fe+U, +Fe-N have been compared to the root exudome of -Fe-N plants (vs. -Fe-N). The treatment +Fe/+Fe-N refers to tomato plants grown under same experimental conditions indicated in this paper under Fe sufficient conditions (+100 µM Fe-EDTA; Lodovici et al., 2024a).

| Compound name | Mass | Formula |  | +Fe/+Fe-N vs -Fe-N | p-value |  | +Fe+A vs -Fe-N | p-value |  | +Fe+Nit vs -Fe-N | p-value |  | +Fe+U vs -Fe-N | p-value |  | +Fe-N vs -Fe-N | p-value |
| --- | --- | --- | --- | --- | --- | --- | --- | --- | --- | --- | --- | --- | --- | --- | --- | --- | --- |
| 7-Hydroxysecoisolariciresinol | 378.1645 | C20 H26 O7 |  | -29.460 | 0.029 |  |  |  |  | -2.139 | 0.015 |  |  |  |  | -1.819 | 0.0235 |
| Feruloyl tartaric acid | 326.0613 | C14 H14 O9 |  | -29.460 | 0.000 |  | -1.526 | 0.02 |  | -2.200 | 0.001 |  | -29.460 | 0.00 |  | -29.460 | 0.0002 |
| p-Coumaroylquinic acid | 338.0998 | C16 H18 O8 |  | -29.460 | 0.001 |  | -0.474 | 0.03 |  | -0.342 | 0.007 |  | -1.513 | 0.00 |  | -1.342 | 0.0012 |
| Rosmarinic acid | 360.0817 | C18 H16 O8 |  | -29.460 | 0.001 |  | -0.487 | 0.03 |  | -0.355 | 0.007 |  | -1.526 | 0.00 |  | -1.355 | 0.0012 |
| avenacin B2 | 1048.524 | C54 H80 O20 |  | -1.630 | 0.047 |  |  |  |  |  |  |  |  |  |  |  |  |
| avenacin A1 | 1093.553 | C55 H83 N O21 |  | -1.376 | 0.014 |  | -1.197 | 0.00 |  |  |  |  |  |  |  | -0.808 | 0.0351 |
| GR24 | 298.0904 | C17 H14 O5 |  | -0.610 | 0.025 |  |  |  |  | -3.413 | 0.000 |  | -2.128 | 0.00 |  | -1.585 | 0.0280 |
| [6]-Gingerol | 294.1808 | C17 H26 O4 |  | 11.132 | 0.039 |  |  |  |  |  |  |  |  |  |  |  |  |
| Pigment A | 609.152 | C31 H29 O13 |  | 11.490 | 0.043 |  |  |  |  |  |  |  |  |  |  |  |  |
| 5-Tricosenylresorcinol | 430.386 | C29 H50 O2 |  | 12.505 | 0.036 |  |  |  |  |  |  |  |  |  |  |  |  |
| 5-Pentacosylresorcinol | 460.4152 | C31 H56 O2 |  | 12.843 | 0.026 |  |  |  |  |  |  |  |  |  |  |  |  |
| 5-Heneicosenylresorcinol | 402.355 | C27 H46 O2 |  | 13.304 | 0.024 |  |  |  |  |  |  |  |  |  |  |  |  |
| brachialactone | 334.2133 | C20 H30 O4 |  | 13.536 | 0.012 |  |  |  |  |  |  |  |  |  |  |  |  |
| Piceatannol | 244.0694 | C14 H12 O4 |  | 14.032 | 0.014 |  |  |  |  |  |  |  |  |  |  |  |  |
| Lutein | 568.4264 | C40 H56 O2 |  | 15.125 | 0.003 |  |  |  |  |  |  |  |  |  |  |  |  |
| Malvidin 3-O-(6''-acetyl-glucoside) | 535.1528 | C25 H27 O13 |  | 15.862 | 0.011 |  |  |  |  |  |  |  |  |  |  |  |  |
| Hesperidin | 610.1769 | C28 H34 O15 |  | 16.146 | 0.004 |  |  |  |  |  |  |  |  |  |  |  |  |
| Peonidin 3-O-rutinoside | 609.1724 | C28 H33 O15 |  | 16.166 | 0.005 |  |  |  |  |  |  |  |  |  |  |  |  |
| Cinnamoyl glucose | 310.112 | C15 H18 O7 |  | 16.453 | 0.001 |  |  |  |  |  |  |  |  |  |  |  |  |
| Cyanidin 3-O-glucosyl-rutinoside | 757.2073 | C33 H41 O20 |  | 17.367 | 0.001 |  |  |  |  |  |  |  |  |  |  |  |  |
| Delphinidin 3-O-rutinoside | 611.1692 | C27 H31 O16 |  | 17.398 | 0.002 |  |  |  |  |  |  |  |  |  |  |  |  |
| luteone | 353.1091 | C20 H17 O6 |  | 19.638 | 0.003 |  |  |  |  |  |  |  |  |  |  |  |  |
| 24-Methylcholestanol ferulate | 578.4291 | C38 H58 O4 |  |  |  |  |  |  |  |  |  |  | 1.878 | 0.02 |  |  |  |
| 5-deoxystrigol | 345.138 | C19 H21 O6 |  |  |  |  | -1.096 | 0.04 |  |  |  |  |  |  |  |  |  |
| 6-Prenylnaringenin | 340.1292 | C20 H20 O5 |  |  |  |  |  |  |  |  |  |  | -1.883 | 0.00 |  |  |  |
| avenacin A2 | 1064.518 | C54 H80 O21 |  |  |  |  | -2.806 | 0.00 |  |  |  |  |  |  |  |  |  |
| Hesperetin | 302.0801 | C16 H14 O6 |  |  |  |  | -1.935 | 0.02 |  |  |  |  |  |  |  |  |  |
| Kaempferol 3-O-glucosyl-rhamnosyl-galactoside | 756.2304 | C33 H40 O20 |  |  |  |  |  |  |  |  |  |  |  |  |  | 0.526 | 0.0201 |
| L-arginino-succinate | 289.1221 | C10 H17 N4 O6 |  |  |  |  |  |  |  |  |  |  |  |  |  | 0.961 | 0.0002 |
| Lariciresinol | 360.1539 | C20 H24 O6 |  |  |  |  | -1.922 | 0.05 |  |  |  |  |  |  |  |  |  |
| Luteolin 7-O-(2-apiosyl-glucoside) | 580.1261 | C26 H28 O15 |  |  |  |  |  |  |  |  |  |  | 2.345 | 0.01 |  |  |  |
| Nobiletin | 402.1329 | C21 H22 O8 |  |  |  |  | -2.878 | 0.00 |  |  |  |  | -1.372 | 0.05 |  |  |  |
| Quercetin 4'-O-glucoside | 384.1222 | C21 H20 O7 |  |  |  |  | -2.878 | 0.00 |  |  |  |  | -1.372 | 0.05 |  |  |  |
| Secoisolariciresinol-sesquilignan | 542.2524 | C30 H38 O9 |  |  |  |  |  |  |  | -2.094 | 0.007 |  |  |  |  |  |  |
| Theaflavin 3,3'-O-digallate | 868.1359 | C43 H32 O20 |  |  |  |  |  |  |  |  |  |  | 1.145 | 0.04 |  |  |  |
| Verbascoside | 624.1977 | C29 H36 O15 |  |  |  |  |  |  |  |  |  |  |  |  |  | 2.070 | 0.0029 |

**Supplementary Table S6. Root exudates by tomato plants after 24 hours from the supply of Fe and N forms.** Value are expressed as LogFC. The root exudome of +Fe/+Fe-N, +Fe+A, +Fe+Nit, +Fe+U, +Fe-N have been compared to the root exudome of -Fe-N plants (vs. -Fe-N). The treatment +Fe/+Fe-N refers to tomato plants grown under same experimental conditions indicated in this paper under Fe sufficient conditions (+100 µM Fe-EDTA; Lodovici et al., 2024a).

| Compound name | Mass | Formula |  | +Fe/+Fe-N | p-value |  | +Fe+A | p-value |  | +Fe+Nit | p-value |  | +Fe+U | p-value |  | +Fe-N | p-value |
| --- | --- | --- | --- | --- | --- | --- | --- | --- | --- | --- | --- | --- | --- | --- | --- | --- | --- |
| L-citrulline | 175.0984 | C6 H13 N3 O3 |  | 6.749 | 0.000 |  |  |  |  |  |  |  |  |  |  |  |  |
| Cyanidin 3-O-sambubioside | 581.1499 | C26 H29 O15 |  | 6.749 | 0.000 |  |  |  |  |  |  |  |  |  |  |  |  |
| Delphinidin 3-O-arabinoside | 435.0874 | C20 H19 O11 |  | 6.749 | 0.000 |  |  |  |  |  |  |  |  |  |  |  |  |
| Theaflavin | 564.1388 | C29 H24 O12 |  | 6.749 | 0.000 |  |  |  |  |  |  |  |  |  |  |  |  |
| Apigenin 7-O-apiosyl-glucoside | 564.1397 | C26 H28 O14 |  | 6.749 | 0.000 |  |  |  |  |  |  |  |  |  |  |  |  |
| 4-methylsulfinylbutyl glucosinolate | 436.0359 | C12 H22 N O10 S3 |  | 6.749 | 0.000 |  |  |  |  |  |  |  |  |  |  |  |  |
| 5-O-Galloylquinic acid | 344.0714 | C14 H16 O10 |  | 6.749 | 0.000 |  |  |  |  |  |  |  |  |  |  |  |  |
| Ellagic acid arabinoside | 434.0443 | C19 H14 O12 |  | 6.749 | 0.000 |  |  |  |  |  |  |  |  |  |  |  |  |
| 5-5'-Dehydrodiferulic acid | 386.0998 | C20 H18 O8 |  | 6.749 | 0.000 |  |  |  |  |  |  |  |  |  |  |  |  |
| Avenanthramide 2f | 329.0817 | C17 H15 N O6 |  | 6.749 | 0.000 |  |  |  |  |  |  |  |  |  |  |  |  |
| Feruloyl tartaric acid | 326.061 | C14 H14 O9 |  | 6.749 | 0.000 |  |  |  |  |  |  |  |  |  |  |  |  |
| 6''-O-Acetylgenistin | 474.1206 | C23 H22 O11 |  | 6.749 | 0.000 |  |  |  |  |  |  |  |  |  |  |  |  |
| Glycitein | 284.0676 | C16 H12 O5 |  | 6.749 | 0.000 |  |  |  |  |  |  |  |  |  |  |  |  |
| aminocyclopropane-1-carboxilic | 101.0492 | C4 H7 N O2 |  | 2.257 | 0.000 |  |  |  |  |  |  |  |  |  |  |  |  |
| L-homoserine | 119.0597 | C4 H9 N O3 |  | 2.257 | 0.000 |  |  |  |  |  |  |  |  |  |  |  |  |
| benzyl-desulfoglucosinolate | 329.1011 | C14 H19 N O6 S |  | 6.749 | 0.000 |  |  |  |  |  |  |  |  |  |  |  |  |
| 1-Acetoxypinoresinol | 416.1512 | C22 H24 O8 |  | 1.289 | 0.000 |  |  |  |  |  |  |  |  |  |  |  |  |
| alpha-aminoadipic acid | 161.0676 | C6 H11 N O4 |  | 2.100 | 0.000 |  |  |  |  |  |  |  |  |  |  |  |  |
| Syringaldehyde | 182.0575 | C9 H10 O4 |  | 2.030 | 0.000 |  |  |  |  |  |  |  |  |  |  |  |  |
| Benzoic acid | 122.0361 | C7 H6 O2 |  | 6.749 | 0.000 |  |  |  |  |  |  |  |  |  |  |  |  |
| Patuletin 3-O-glucosyl-(1-6)-[apiosyl(1-2)]-glucoside | 788.1903 | C33 H40 O22 |  | 6.749 | 0.000 |  |  |  |  |  |  |  |  |  |  |  |  |
| Pelargonidin 3-O-arabinoside | 403.1019 | C20 H19 O9 |  | 1.777 | 0.000 |  |  |  |  |  |  |  |  |  |  |  |  |
| Kaempferol 3,7,4'-O-triglucoside | 772.2151 | C33 H40 O21 |  | 6.749 | 0.000 |  |  |  |  |  |  |  |  |  |  |  |  |
| p-Coumaric acid | 164.0467 | C9 H8 O3 |  | 1.962 | 0.000 |  |  |  |  |  |  |  |  |  |  |  |  |
| 6-methylthiohexyldesulfoglucosinolate | 369.1311 | C14 H27 N O6 S2 |  | -1.524 | 0.000 |  |  |  |  |  |  |  |  |  |  |  |  |
| Curcumin | 368.1279 | C21 H20 O6 |  | -1.492 | 0.001 |  |  |  |  |  |  |  |  |  |  |  |  |
| 6-Geranylnaringenin | 408.194 | C25 H28 O5 |  | -2.542 | 0.001 |  |  |  |  |  |  |  |  |  |  | -1.588 | 0.008 |
| Lycopene | 536.4305 | C40 H56 |  | -2.503 | 0.002 |  |  |  |  |  |  |  |  |  |  |  |  |
| Palmitic acid | 256.2405 | C16 H32 O2 |  | -1.912 | 0.003 |  |  |  |  |  |  |  |  |  |  |  |  |
| (+)-Catechin | 290.0771 | C15 H14 O6 |  | 6.749 | 0.004 |  |  |  |  |  |  |  |  |  |  |  |  |
| Myricetin 3-O-arabinoside | 450.0799 | C20 H18 O12 |  | -2.383 | 0.008 |  |  |  |  |  |  |  |  |  |  |  |  |
| Sinensetin | 372.1262 | C20 H20 O7 |  | -2.127 | 0.010 |  |  |  |  |  |  |  |  |  |  |  |  |
| Theaflavin 3,3'-O-digallate | 868.1359 | C43 H32 O20 |  | -2.462 | 0.011 |  |  |  |  |  |  |  |  |  |  |  |  |
| Quercetin 3-O-xylosyl-glucuronide | 770.0616 | C26 H26 O27 |  | -1.923 | 0.016 |  |  |  |  |  |  |  |  |  |  |  |  |
| isosakuranetin | 285.0827 | C16 H13 O5 |  | 1.813 | 0.020 |  |  |  |  |  |  |  |  |  |  |  |  |
| Pinoresinol | 358.1425 | C20 H22 O6 |  | 2.127 | 0.028 |  |  |  |  |  |  |  |  |  |  |  |  |
| 24-Methylenecholestanol ferulate | 577.4216 | C38 H57 O4 |  | -1.487 | 0.043 |  |  |  |  |  |  |  |  |  |  |  |  |
| Lariciresinol | 360.1539 | C20 H24 O6 |  | -1.749 | 0.009 |  | -2.628 | 0.00 |  |  |  |  |  |  |  | -1.399 | 0.048 |
| Secoisolariciresinol-sesquilignan | 542.2567 | C30 H38 O9 |  |  |  |  | 2.406 | 0.02 |  |  |  |  |  |  |  |  |  |
| Sinapine | 310.172 | C16 H24 N O5 |  |  |  |  |  |  |  | 11.67 | 0.00 |  |  |  |  |  |  |
| 5-methylthiopentyldesulfoglucosinolate | 355.1178 | C13 H25 N O6 S2 |  | 0.984 | 0.006 |  |  |  |  | 1.46 | 0.01 |  |  |  |  |  |  |
| 7-Oxomatairesinol | 373.1283 | C20 H21 O7 |  |  |  |  |  |  |  | 1.46 | 0.02 |  |  |  |  |  |  |
| Pterostilbene | 256.1057 | C16 H16 O3 |  |  |  |  |  |  |  | 17.35 | 0.03 |  |  |  |  |  |  |
| Quercetin 4'-O-glucoside | 384.1222 | C21 H20 O7 |  | -4.499 | 0.000 |  | -4.148 | 0.00 |  |  |  |  | -2.87 | 0.00 |  | -2.208 | 0.005 |
| Nobiletin | 402.1329 | C21 H22 O8 |  | -4.354 | 0.001 |  | -4.217 | 0.00 |  |  |  |  | -2.87 | 0.00 |  | -2.211 | 0.011 |
| Arachidic acid | 312.3067 | C20 H40 O2 |  | -6.749 | 0.001 |  | -6.749 | 0.00 |  | -6.75 | 0.001 |  | -0.68 | 0.00 |  | -1.741 | 0.000 |
| Campesteryl ferulate | 592.4126 | C38 H56 O5 |  | -6.749 | 0.010 |  | -6.749 | 0.01 |  |  |  |  | -1.34 | 0.01 |  |  |  |

**Supplementary Table S7. List of the fifteen most downregulated DEGs for each comparison.**

| IDENTIFIER | DESCRIPTION | Log2FC (+Fe+U vs -Fe-N) | Log2FC (+Fe+A vs -Fe-N) | Log2FC (+Fe+Nit vs -Fe-N) | Log2FC (+Fe-N vs -Fe-N) |
| --- | --- | --- | --- | --- | --- |
|  |  |  |  |  |  |
| **Fifteen most downregulated DEGs by +Fe+U vs -Fe-N** | | | | | |
| Solyc06g074710.1.1 | Agmatine hydroxycinnamoyltransferase 1 OS=Oryza sativa subsp. japonica (sp\|q7xpk7\|aht1_orysj : 458.0) & Enzyme classification.EC_2 transferases.EC_2.3 acyltransferase(50.2.3 : 35.2) | -3.48 | -4.40 | 0.00 | -2.25 |
| Solyc12g099180.3.1 | poly(A) RNA polymerase | -3.49 |  | -1.89 |  |
| Solyc05g011890.1.1 | steroid sulphotransferase | -3.52 |  |  | -2.06 |
| Solyc12g008380.1.1 | no hits & | -3.53 | -3.28 | -2.89 | 0.00 |
| Solyc08g068630.4.1 | Histidine decarboxylase OS=Solanum lycopersicum (sp\|p54772\|dchs_sollc : 585.0) | -3.56 | -2.62 |  |  |
| Solyc11g012980.1.1 | Ethylene-responsive transcription factor ERF014 OS=Arabidopsis thaliana (sp\|q9lpe8\|erf14_arath : 110.0) | -3.65 | -4.34 | -3.46 | -2.89 |
| Solyc10g018150.2.1 | Enzyme classification.EC_1 oxidoreductases.EC_1.14 oxidoreductase acting on paired donor with incorporation or reduction of molecular oxygen(50.1.13 : 351.6) & 3,9-dihydroxypterocarpan 6A-monooxygenase OS=Glycine max (sp\|q42798\|c93a1_soybn : 349.0) | -3.84 | -2.99 | -3.61 | -2.24 |
| Solyc08g079300.3.1 | Enzyme classification.EC_1 oxidoreductases.EC_1.14 oxidoreductase acting on paired donor with incorporation or reduction of molecular oxygen(50.1.13 : 396.4) & Flavonoid 3-monooxygenase OS=Petunia hybrida (sp\|q9sbq9\|f3ph_pethy : 337.0) | -3.88 | -5.51 | -3.41 | -2.48 |
| Solyc07g025140.4.1 | Protein JINGUBANG OS=Arabidopsis thaliana (sp\|o48716\|jgb_arath : 533.0) | -4.04 | -1.11 | -2.74 | 0.00 |
| Solyc09g065750.3.1 | carotenoid isomerase (DWARF27) | -4.08 | -3.78 | -4.72 | -1.86 |
| Solyc01g079660.2.1 | no hits & | -4.08 | -4.77 | -4.62 | -4.04 |
| Solyc09g007940.3.1 | adenosine kinase | -4.08 | -2.12 | -2.40 | -1.89 |
| Solyc12g017240.2.1 | Xyloglucan endotransglucosylase/hydrolase protein 15 OS=Arabidopsis thaliana (sp\|q38911\|xth15_arath : 365.0) & Enzyme classification.EC_2 transferases.EC_2.4 glycosyltransferase(50.2.4 : 313.6) | -4.08 |  | -3.51 | -3.36 |
| Solyc01g079180.4.1 | pectin methylesterase | -4.18 | -3.42 | -1.43 | 0.00 |
| Solyc03g124110.2.1 | transcription factor (DREB) | -4.70 |  | -3.39 | -4.61 |
|  |  |  |  |  |  |
| **Fifteen most downregulated DEGs by +Fe+A vs -Fe-N** | | | | | |
| Solyc09g065750.3.1 | carotenoid isomerase (DWARF27) | -4.08 | -3.78 | -4.72 | -1.86 |
| Solyc11g072560.3.1 | anion transporter (NRT1/PTR) | -3.44 | -3.89 | -1.33 | 0.00 |
| Solyc01g108240.3.1 | transcription factor (ERF) | -3.38 | -4.02 | -3.01 | -3.52 |
| Solyc03g093080.3.1 | Probable xyloglucan endotransglucosylase/hydrolase protein 23 OS=Arabidopsis thaliana (sp\|q38910\|xth23_arath : 416.0) & Enzyme classification.EC_2 transferases.EC_2.4 glycosyltransferase(50.2.4 : 326.8) | -3.13 | -4.08 | -2.08 | -2.06 |
| Solyc03g093120.5.1.1 | Probable xyloglucan endotransglucosylase/hydrolase protein 23 OS=Arabidopsis thaliana (sp\|q38910\|xth23_arath : 399.0) & Enzyme classification.EC_2 transferases.EC_2.4 glycosyltransferase(50.2.4 : 319.1) | -3.32 | -4.13 | -2.48 | -2.08 |
| Solyc12g044190.3.1 | Probable disease resistance protein At1g61180 OS=Arabidopsis thaliana (sp\|q940k0\|drl15_arath : 214.0) |  | -4.14 | -1.51 | -2.22 |
| Solyc03g026280.3.1 | transcription factor (DREB) | -3.12 | -4.26 | -3.79 | -3.25 |
| Solyc05g055220.1.1 | transcription factor (OFP) | -2.98 | -4.26 | -2.13 | -1.18 |
| Solyc02g078400.3.1 | allantoinase | -1.46 | -4.29 | -1.66 | 0.00 |
| Solyc12g005450.1.1 | receptor-like protein kinase (RLCK-XIII) | -1.99 | -4.29 | -2.62 | -2.51 |
| Solyc11g012980.1.1 | Ethylene-responsive transcription factor ERF014 OS=Arabidopsis thaliana (sp\|q9lpe8\|erf14_arath : 110.0) | -3.65 | -4.34 | -3.46 | -2.89 |
| Solyc06g074710.1.1 | Agmatine hydroxycinnamoyltransferase 1 OS=Oryza sativa subsp. japonica (sp\|q7xpk7\|aht1_orysj : 458.0) & Enzyme classification.EC_2 transferases.EC_2.3 acyltransferase(50.2.3 : 35.2) | -3.48 | -4.40 |  | -2.25 |
| Solyc01g079660.2.1 | no hits & | -4.08 | -4.77 | -4.62 | -4.04 |
| Solyc03g093110.3.1 | Probable xyloglucan endotransglucosylase/hydrolase protein 23 OS=Arabidopsis thaliana (sp\|q38910\|xth23_arath : 400.0) & Enzyme classification.EC_2 transferases.EC_2.4 glycosyltransferase(50.2.4 : 319.9) | -3.32 | -5.22 | -3.12 | -2.72 |
| Solyc08g079300.3.1 | Enzyme classification.EC_1 oxidoreductases.EC_1.14 oxidoreductase acting on paired donor with incorporation or reduction of molecular oxygen(50.1.13 : 396.4) & Flavonoid 3-monooxygenase OS=Petunia hybrida (sp\|q9sbq9\|f3ph_pethy : 337.0) | -3.88 | -5.51 | -3.41 | -2.48 |
|  |  |  |  |  |  |
| **Fifteen most downregulated DEGs by +Fe+Nit vs -Fe-N** | | | | | |
| Solyc10g085500.2.1 | Cytochrome P450 76A2 OS=Solanum melongena (sp\|p37122\|c76a2_solme : 584.0) & Enzyme classification.EC_1 oxidoreductases.EC_1.14 oxidoreductase acting on paired donor with incorporation or reduction of molecular oxygen(50.1.13 : 381.8) | -1.82 | -1.80 | -3.26 | -1.30 |
| Solyc12g056180.3.1 | 1-aminocyclopropane-1-carboxylate (ACC) synthase | -1.91 | -3.21 | -3.28 | -2.91 |
| Solyc03g124110.2.1 | transcription factor (DREB) | -4.70 |  | -3.39 | -4.61 |
| Solyc08g079300.3.1 | Enzyme classification.EC_1 oxidoreductases.EC_1.14 oxidoreductase acting on paired donor with incorporation or reduction of molecular oxygen(50.1.13 : 396.4) & Flavonoid 3-monooxygenase OS=Petunia hybrida (sp\|q9sbq9\|f3ph_pethy : 337.0) | -3.88 | -5.51 | -3.41 | -2.48 |
| Solyc11g012980.1.1 | Ethylene-responsive transcription factor ERF014 OS=Arabidopsis thaliana (sp\|q9lpe8\|erf14_arath : 110.0) | -3.65 | -4.34 | -3.46 | -2.89 |
| Solyc12g017240.2.1 | Xyloglucan endotransglucosylase/hydrolase protein 15 OS=Arabidopsis thaliana (sp\|q38911\|xth15_arath : 365.0) & Enzyme classification.EC_2 transferases.EC_2.4 glycosyltransferase(50.2.4 : 313.6) | -4.08 |  | -3.51 | -3.36 |
| Solyc10g018150.2.1 | Enzyme classification.EC_1 oxidoreductases.EC_1.14 oxidoreductase acting on paired donor with incorporation or reduction of molecular oxygen(50.1.13 : 351.6) & 3,9-dihydroxypterocarpan 6A-monooxygenase OS=Glycine max (sp\|q42798\|c93a1_soybn : 349.0) | -3.84 | -2.99 | -3.61 | -2.24 |
| Solyc03g026280.3.1 | transcription factor (DREB) | -3.12 | -4.26 | -3.79 | -3.25 |
| Solyc06g048750.3.1 | no hits | -1.43 |  | -3.80 |  |
| Solyc12g009240.1.1 | transcription factor (DREB) | -2.16 | -3.55 | -4.00 | -2.87 |
| Solyc03g026270.3.1 | transcription factor (DREB) |  |  | -4.19 |  |
| Solyc04g079510.2.1 | anion transporter (NRT1/PTR) | -3.00 | -3.33 | -4.28 | -4.50 |
| Solyc01g079660.2.1 | no hits | -4.08 | -4.77 | -4.62 | -4.04 |
| Solyc09g065750.3.1 | carotenoid isomerase (DWARF27) | -4.08 | -3.78 | -4.72 | -1.86 |
| Solyc03g078500.3.1 | 7-deoxyloganetin glucosyltransferase OS=Catharanthus roseus (sp\|f8wls6\|ugt6_catro : 582.0) & Enzyme classification.EC_2 transferases.EC_2.4 glycosyltransferase(50.2.4 : 262.2) | -2.98 | -3.44 | -5.12 | -2.37 |
|  |  |  |  |  |  |
| **Fifteen most downregulated DEGs by +Fe-N vs -Fe-N** | | | | | |
| Solyc03g093110.3.1 | Probable xyloglucan endotransglucosylase/hydrolase protein 23 OS=Arabidopsis thaliana (sp\|q38910\|xth23_arath : 400.0) & Enzyme classification.EC_2 transferases.EC_2.4 glycosyltransferase(50.2.4 : 319.9) | -3.32 | -5.22 | -3.12 | -2.72 |
| Solyc08g081500.3.1 | transcription factor (MYB) | -1.74 |  |  | -2.81 |
| Solyc07g040960.1.1 | Protein ALP1-like OS=Arabidopsis thaliana (sp\|q9m2u3\|alpl_arath : 155.0) | -2.02 | -3.32 | -2.85 | -2.84 |
| Solyc12g009240.1.1 | transcription factor (DREB) | -2.16 | -3.55 | -4.00 | -2.87 |
| Solyc11g012980.1.1 | Ethylene-responsive transcription factor ERF014 OS=Arabidopsis thaliana (sp\|q9lpe8\|erf14_arath : 110.0) | -3.65 | -4.34 | -3.46 | -2.89 |
| Solyc12g056180.3.1 | 1-aminocyclopropane-1-carboxylate (ACC) synthase | -1.91 | -3.21 | -3.28 | -2.91 |
| Solyc11g071760.3.1 | Calcium-binding protein CML37 OS=Arabidopsis thaliana (sp\|q9fih9\|cml37_arath : 148.0) | -2.85 | -2.90 | -2.75 | -2.96 |
| Solyc08g066720.3.1 | Carotenoid 9,10(9,10)-cleavage dioxygenase 1 OS=Pisum sativum (sp\|q8lp17\|ccd1_pea : 194.0) |  | -2.85 | -2.19 | -3.12 |
| Solyc03g026280.3.1 | transcription factor (DREB) | -3.12 | -4.26 | -3.79 | -3.25 |
| Solyc12g017240.2.1 | Xyloglucan endotransglucosylase/hydrolase protein 15 OS=Arabidopsis thaliana (sp\|q38911\|xth15_arath : 365.0) & Enzyme classification.EC_2 transferases.EC_2.4 glycosyltransferase(50.2.4 : 313.6) | -4.08 |  | -3.51 | -3.36 |
| Solyc02g064980.1.1 | protein kinase (MAP3K-MEKK) | -3.04 | -Inf | -2.08 | -3.37 |
| Solyc01g108240.3.1 | transcription factor (ERF) | -3.38 | -4.02 | -3.01 | -3.52 |
| Solyc01g079660.2.1 | no hits | -4.08 | -4.77 | -4.62 | -4.04 |
| Solyc04g079510.2.1 | anion transporter (NRT1/PTR) | -3.00 | -3.33 | -4.28 | -4.50 |
| Solyc03g124110.2.1 | transcription factor (DREB) | -4.70 |  | -3.39 | -4.61 |

**Supplementary Table S8. List of the fifteen most upregulated DEGs for each comparison.**

| IDENTIFIER | DESCRIPTION | Log2FC (+Fe+U vs -Fe-N) | Log2FC (+Fe+A vs -Fe-N) | Log2FC (+Fe+Nit vs -Fe-N) | Log2FC (+Fe-N vs -Fe-N) |
| --- | --- | --- | --- | --- | --- |
|  |  |  |  |  |  |
| **Fifteen most overexpressed DEGs by +Fe+U vs -Fe-N** | | | | | |
| Solyc06g050870.3.1 | no hits | 8.87 | 3.71 |  |  |
| Solyc05g053860.4.1 | organic cation transporter (OCT) | 8.44 | 4.83 |  |  |
| Solyc07g026650.3.1 | 1-aminocyclopropane-1-carboxylate (ACC) oxidase | 7.08 | 2.95 |  |  |
| Solyc02g089140.3.1 | no hits | 6.94 |  |  |  |
| Solyc07g063640.1.1 | no hits | 6.01 | 3.52 |  |  |
| Solyc11g008680.2.1 | stearoyl-ACP desaturase | 5.68 | 1.60 |  |  |
| Solyc02g090120.1.1 | no hits | 5.66 | 2.88 |  |  |
| Solyc02g077240.4.1 | pyruvate decarboxylase | 5.54 | 2.28 |  |  |
| Solyc10g005550.3.1 | transcription factor (MYB) | 5.37 | 4.85 | 5.69 |  |
| Solyc05g047530.3.1 | Enzyme classification.EC_1 oxidoreductases.EC_1.14 oxidoreductase acting on paired donor with incorporation or reduction of molecular oxygen(50.1.13 : 788.2) & Cytochrome P450 CYP73A100 OS=Panax ginseng (sp\|h2dh22\|c7a10_pangi : 741.0) | 5.19 | 2.24 |  | 1.38 |
| Solyc12g011060.3.1 | no hits & | 5.11 | 4.70 | 6.30 | 4.99 |
| Solyc01g104820.4.1 | iron cation transporter (VTL) | 5.09 | 5.56 | 5.22 | 5.04 |
| Solyc02g069190.4.1 | metal cation transporter (ZIP) | 5.06 | 2.36 | 4.01 | 2.30 |
| Solyc12g088130.3.1 | transcription factor (bHLH) | 5.05 |  |  |  |
| Solyc09g015000.4.1 | 18.2 kDa class I heat shock protein OS=Medicago sativa (sp\|p27880\|hsp12_medsa : 179.0) | 4.91 |  |  |  |
|  |  |  |  |  |  |
| **Fifteen most overexpressed DEGs by +Fe+A vs -Fe-N** | | | | | |
| Solyc04g077990.3.1 | transcription factor (AS2/LOB) | 4.05 | 5.59 |  |  |
| Solyc01g104820.4.1 | iron cation transporter (VTL) | 5.09 | 5.56 | 5.22 | 5.04 |
| Solyc10g005550.3.1 | transcription factor (MYB) | 5.37 | 4.85 | 5.69 |  |
| Solyc05g053860.4.1 | organic cation transporter (OCT) | 8.44 | 4.83 |  |  |
| Solyc12g011060.3.1 | no hits | 5.11 | 4.70 | 6.30 | 4.99 |
| Solyc06g007180.3.1 | glutamine-dependent asparagine synthetase | 2.76 | 4.19 |  |  |
| Solyc10g044447.1.1 | feruloyl-CoA 6-hydroxylase | 4.44 | 3.82 | 5.62 | 3.71 |
| Solyc07g049560.3.1 | atypical dual-specificity phosphatase (PFA-DSP) | 3.05 | 3.78 | 2.78 | 1.30 |
| Solyc06g050870.3.1 | no hits & | 8.87 | 3.71 |  |  |
| Solyc12g088970.1.1 | Cytochrome P450 82C4 OS=Arabidopsis thaliana (sp\|q9sz46\|c82c4_arath : 695.0) & Enzyme classification.EC_1 oxidoreductases.EC_1.14 oxidoreductase acting on paired donor with incorporation or reduction of molecular oxygen(50.1.13 : 331.3) | 3.91 | 3.69 | 4.29 | 3.55 |
| Solyc05g009320.4.1 | transcription factor (AS2/LOB) | 2.90 | 3.59 |  |  |
| Solyc01g005900.2.1 | amino acid transporter (LAT) | 3.50 | 3.56 | 4.66 | 3.66 |
| Solyc07g063640.1.1 | no hits & | 6.01 | 3.52 |  |  |
| Solyc01g104810.2.1 | iron cation transporter (VTL) | 3.38 | 3.49 | 3.02 | 2.71 |
| Solyc02g063410.3.1 | Uncharacterized protein At5g65660 OS=Arabidopsis thaliana (sp\|q9lsk9\|y5566_arath : 130.0) | 3.19 | 3.43 | 4.99 | 3.52 |
|  |  |  |  |  |  |
| **Fifteen most overexpressed DEGs by +Fe+Nit vs -Fe-N** | | | | | |
| Solyc12g011060.3.1 | no hits | 5.11 | 4.70 | 6.30 | 4.99 |
| Solyc08g007430.2.1 | anion transporter (NRT1/PTR) |  | 1.93 | 5.75 |  |
| Solyc10g005550.3.1 | transcription factor (MYB) | 5.37 | 4.85 | 5.69 |  |
| Solyc10g044447.1.1 | feruloyl-CoA 6-hydroxylase | 4.44 | 3.82 | 5.62 | 3.71 |
| Solyc01g104820.4.1 | iron cation transporter (VTL) | 5.09 | 5.56 | 5.22 | 5.04 |
| Solyc02g063410.3.1 | Uncharacterized protein At5g65660 OS=Arabidopsis thaliana (sp\|q9lsk9\|y5566_arath : 130.0) | 3.19 | 3.43 | 4.99 | 3.52 |
| Solyc01g005900.2.1 | amino acid transporter (LAT) | 3.50 | 3.56 | 4.66 | 3.66 |
| Solyc12g088970.1.1 | Cytochrome P450 82C4 OS=Arabidopsis thaliana (sp\|q9sz46\|c82c4_arath : 695.0) & Enzyme classification.EC_1 oxidoreductases.EC_1.14 oxidoreductase acting on paired donor with incorporation or reduction of molecular oxygen(50.1.13 : 331.3) | 3.91 | 3.69 | 4.29 | 3.55 |
| Solyc10g032565.1.1 | feruloyl-CoA 6-hydroxylase | 2.95 | 2.19 | 4.24 | 2.69 |
| Solyc07g049655.1.1 | Benzyl alcohol O-benzoyltransferase OS=Nicotiana tabacum (sp\|q8gt20\|bebt_tobac : 450.0) |  | 2.09 | 4.05 | 2.30 |
| Solyc02g069190.4.1 | metal cation transporter (ZIP) | 5.06 | 2.36 | 4.01 | 2.30 |
| Solyc11g045410.1.1 | no hits & | -Inf |  | 3.95 |  |
| Solyc01g090350.3.1 | Non-specific lipid-transfer protein A OS=Ricinus communis (sp\|p10973\|nltpa_ricco : 109.0) |  |  | 3.87 |  |
| Solyc01g094910.3.1 | iron uptake Fe(III)-chelate reductase | 2.91 | 2.85 | 3.78 | 2.32 |
| Solyc10g081780.3.1 | RING-H2 finger protein ATL70 OS=Arabidopsis thaliana (sp\|q8rx29\|atl70_arath : 118.0) | 4.28 | 3.32 | 3.77 | 3.46 |
|  |  |  |  |  |  |
| **Fifteen most overexpressed DEGs by +Fe-N vs -Fe-N** | | | | | |
| Solyc00g500208.1.1 | apoprotein PsaB of PS-I complex |  |  |  | 5.06 |
| Solyc01g104820.4.1 | iron cation transporter (VTL) | 5.09 | 5.56 | 5.22 | 5.04 |
| Solyc12g011060.3.1 | no hits & | 5.11 | 4.70 | 6.30 | 4.99 |
| Solyc00g500063.1.1 | subunit beta of peripheral CF1 subcomplex of ATP synthase complex | 1.34 |  |  | 4.90 |
| Solyc10g044447.1.1 | feruloyl-CoA 6-hydroxylase | 4.44 | 3.82 | 5.62 | 3.71 |
| Solyc01g005900.2.1 | amino acid transporter (LAT) | 3.50 | 3.56 | 4.66 | 3.66 |
| Solyc12g088970.1.1 | Cytochrome P450 82C4 OS=Arabidopsis thaliana (sp\|q9sz46\|c82c4_arath : 695.0) & Enzyme classification.EC_1 oxidoreductases.EC_1.14 oxidoreductase acting on paired donor with incorporation or reduction of molecular oxygen(50.1.13 : 331.3) | 3.91 | 3.69 | 4.29 | 3.55 |
| Solyc02g063410.3.1 | Uncharacterized protein At5g65660 OS=Arabidopsis thaliana (sp\|q9lsk9\|y5566_arath : 130.0) | 3.19 | 3.43 | 4.99 | 3.52 |
| Solyc10g081780.3.1 | RING-H2 finger protein ATL70 OS=Arabidopsis thaliana (sp\|q8rx29\|atl70_arath : 118.0) | 4.28 | 3.32 | 3.77 | 3.46 |
| Solyc02g062300.3.1 | BURP domain-containing protein 3 OS=Oryza sativa subsp. japonica (sp\|q942d4\|burp3_orysj : 177.0) | 3.98 |  |  | 3.13 |
| Solyc11g045520.2.1 | scopoletin 8-hydroxylase | 2.93 | 3.20 | 3.75 | 2.81 |
| Solyc10g008700.3.1 | transcription factor (MYB) | 2.20 |  | 2.46 | 2.76 |
| Solyc06g059840.4.1 | subunit alpha of E1 2-oxoisovalerate dehydrogenase subcomplex | 1.94 | 2.83 | 3.75 | 2.75 |
| Solyc01g104810.2.1 | iron cation transporter (VTL) | 3.38 | 3.49 | 3.02 | 2.71 |
| Solyc06g008050.4.1 | flavin monooxygenase (YUCCA) | 3.38 | 2.32 | 3.17 | 2.71 |

**Supplementary Figures**

**
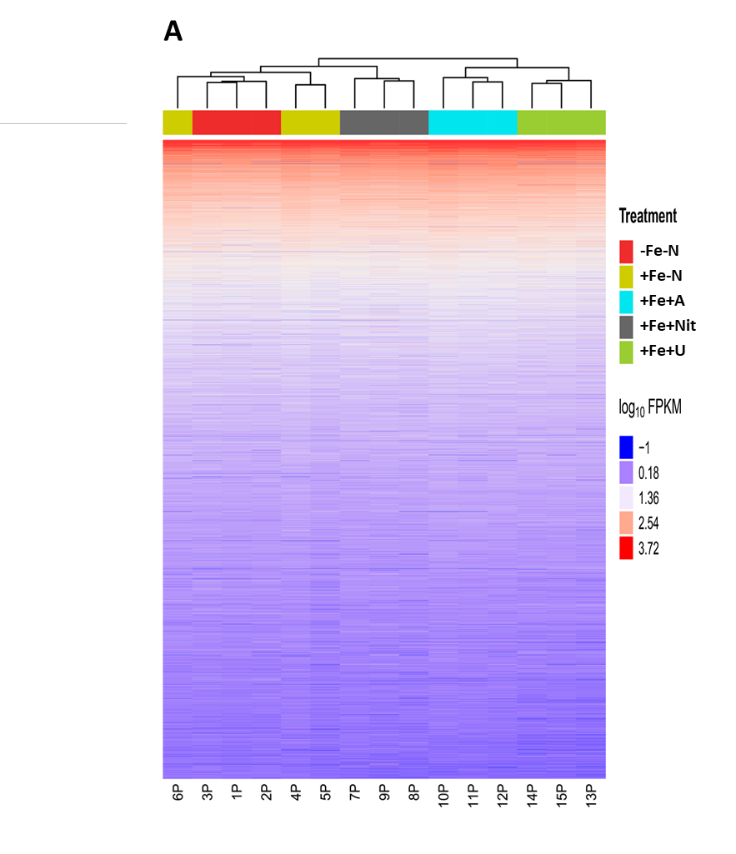

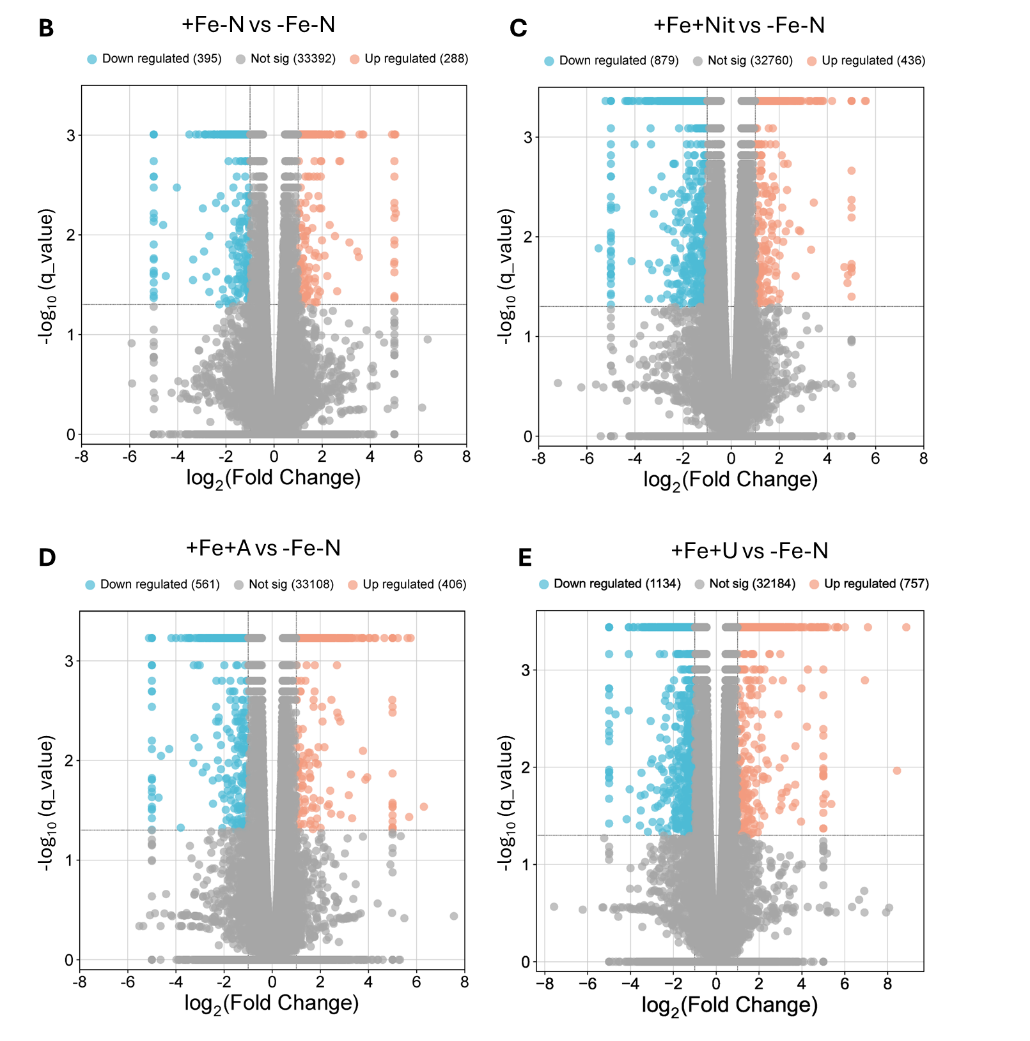
**

**Supplementary Figure S1.** The root transcriptomic profiles are visualized as heatmap with dendrogram showing clustering of samples (data expressed as Log_10_FPKM, **A**). The volcano plots indicated the distribution of significant DEGs (Log_2_|FC| ≥ 1.00, N = 3, q-value ≤0.05) in the four comparisons: +Fe-N vs -Fe-N (**B**); +Fe+Nit vs -Fe-N (**C**); +Fe+A vs -Fe-N (**D**); +Fe+U vs -Fe-N (**E**).

**
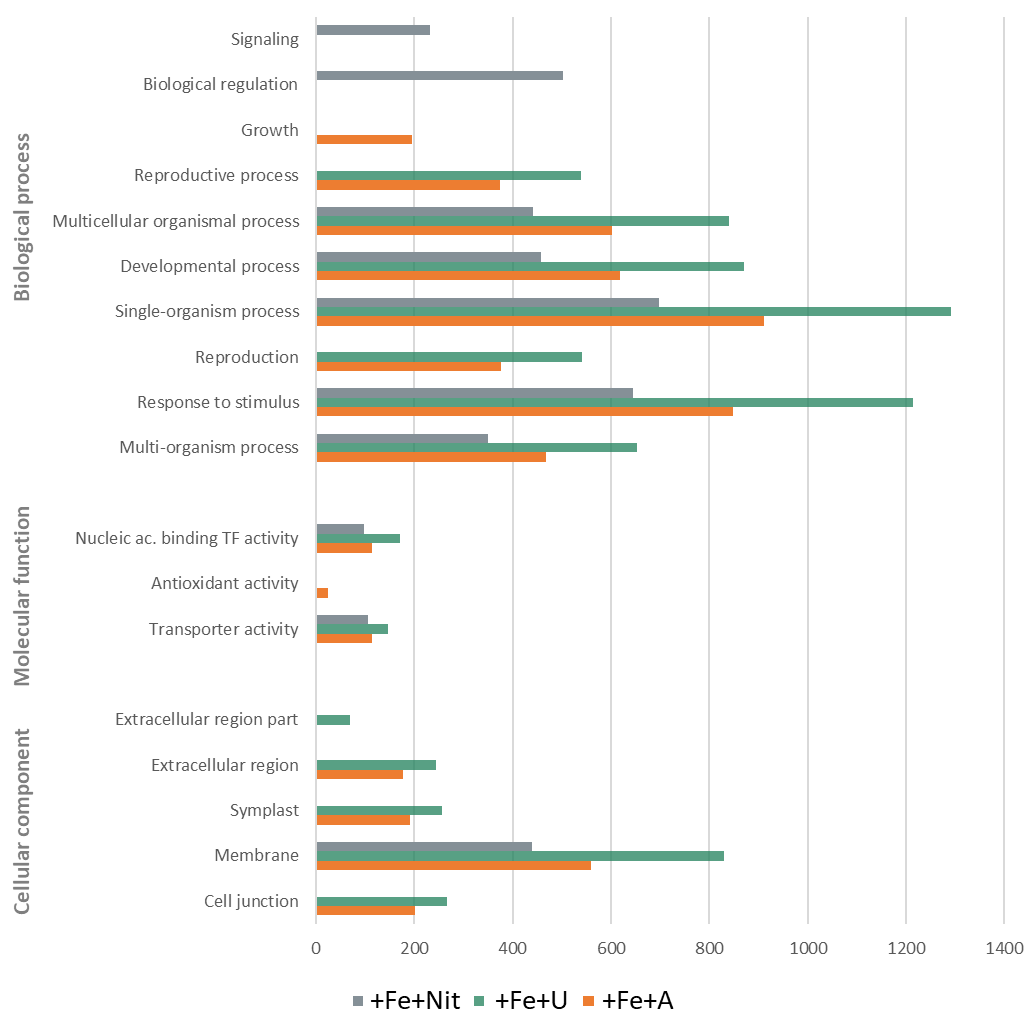
**

**Supplementary Figure S2**. GO enrichment analyses of modulated transcripts performed on AgriGO of the following comparisons: +Fe+Nit, +Fe+U, +Fe+A vs -Fe-N.

**
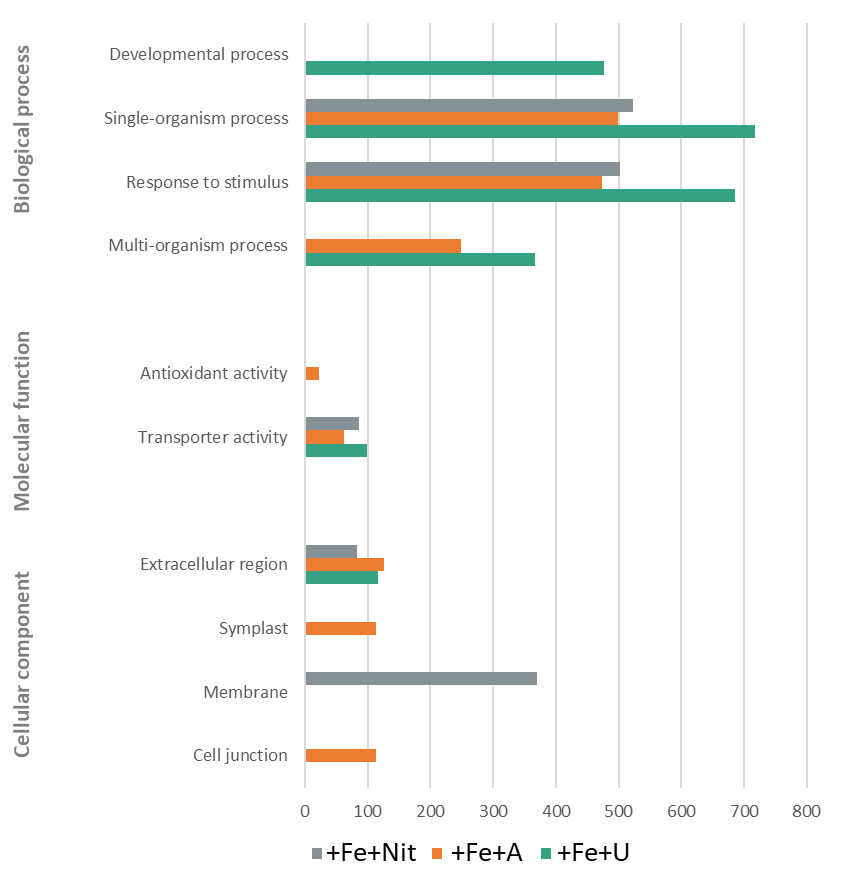
**

**Supplementary Figure S3**. GO enrichment analyses of modulated transcripts performed on AgriGO of the following comparisons: +Fe+Nit, +Fe+U, +Fe+A vs +Fe-N.

**
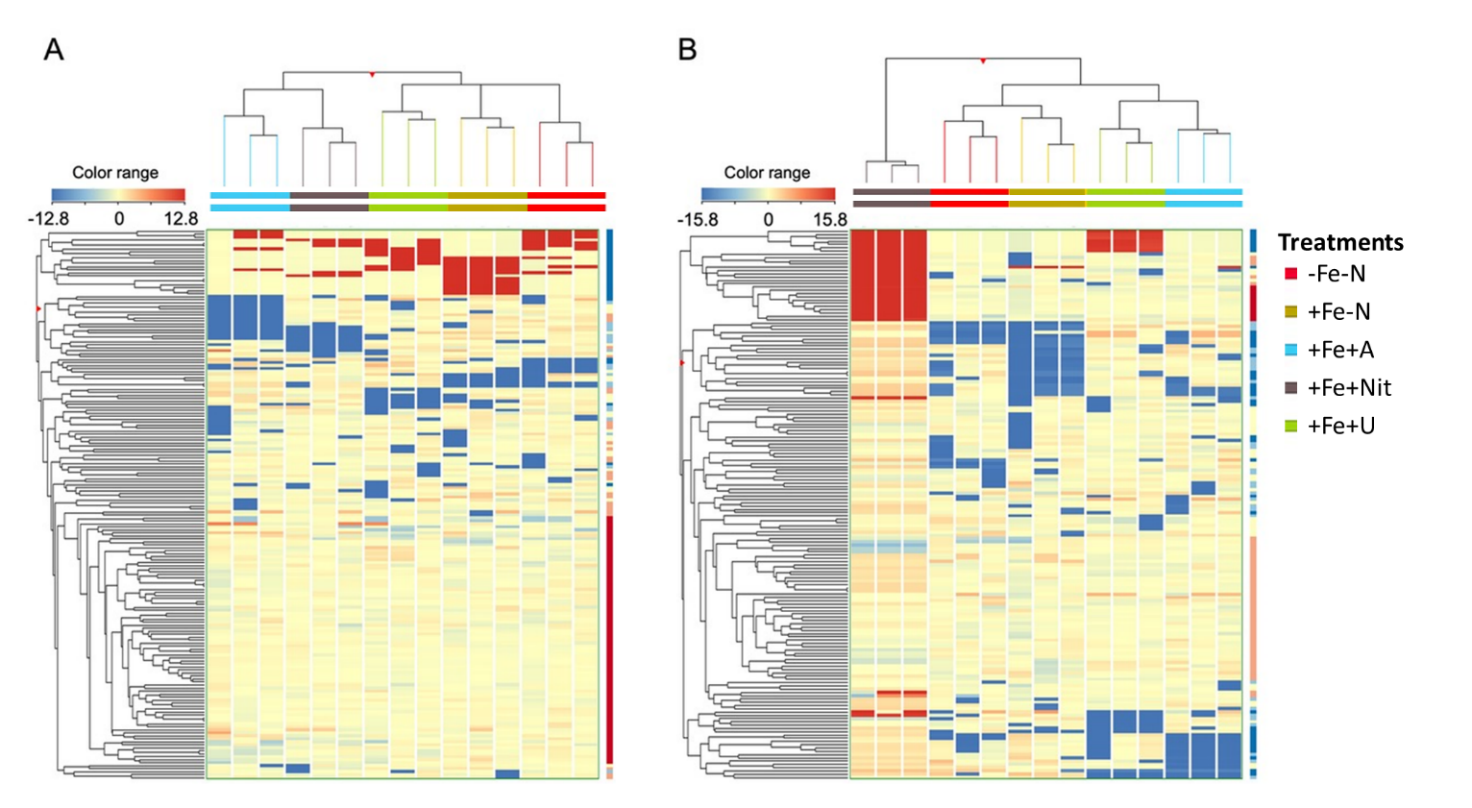
**

**Supplementary Figure S4**. Hierarchical Cluster Analysis of metabolites in the root exudates collected after 4 hours (A) or after 24 hours (B). Color Range refers to the fold change values of metabolites detected in the root exudates in comparison to +Fe/+Fe-N (plants grown under Fe sufficient conditions +100 µM Fe-EDTA).


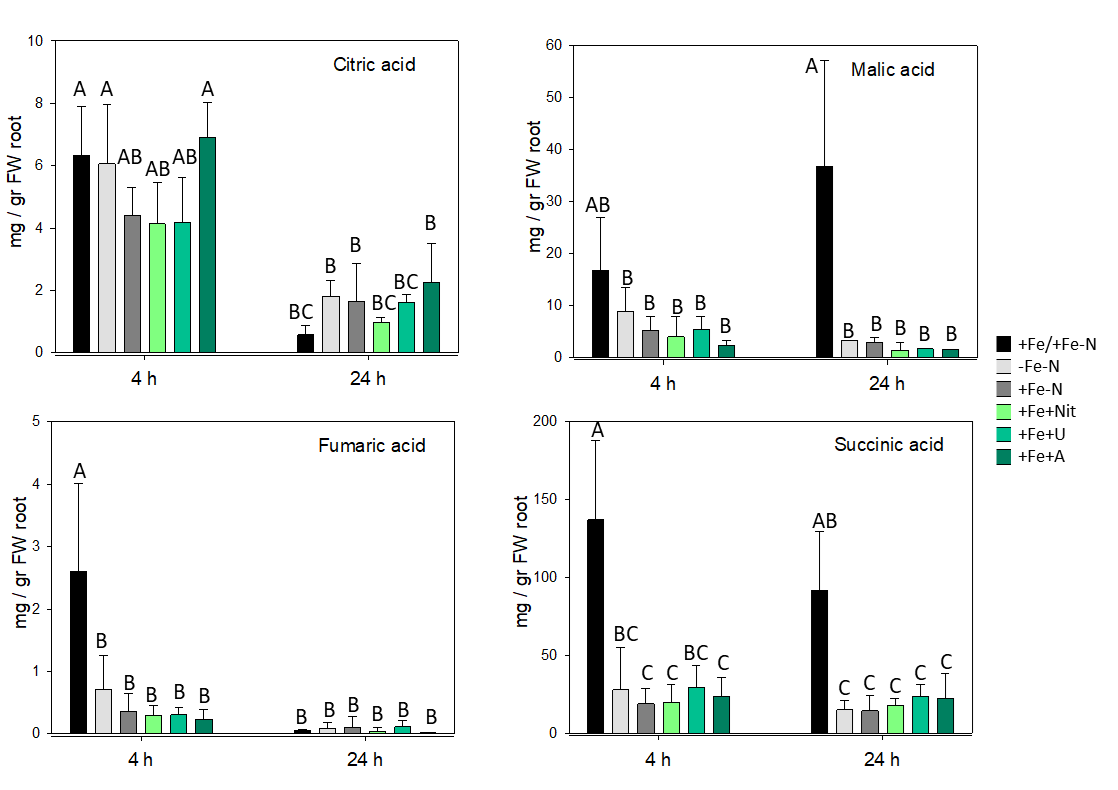


**Supplementary Figure S5**. Organic acid concentration in root exudates of tomato plants after 4 and 24 hours of treatment (Holm-Siddak method, one way-ANOVA, N=3, p<0.05). The treatment +Fe/+Fe-N refers to tomato plants grown under Fe sufficient conditions (+100 µM Fe-EDTA).


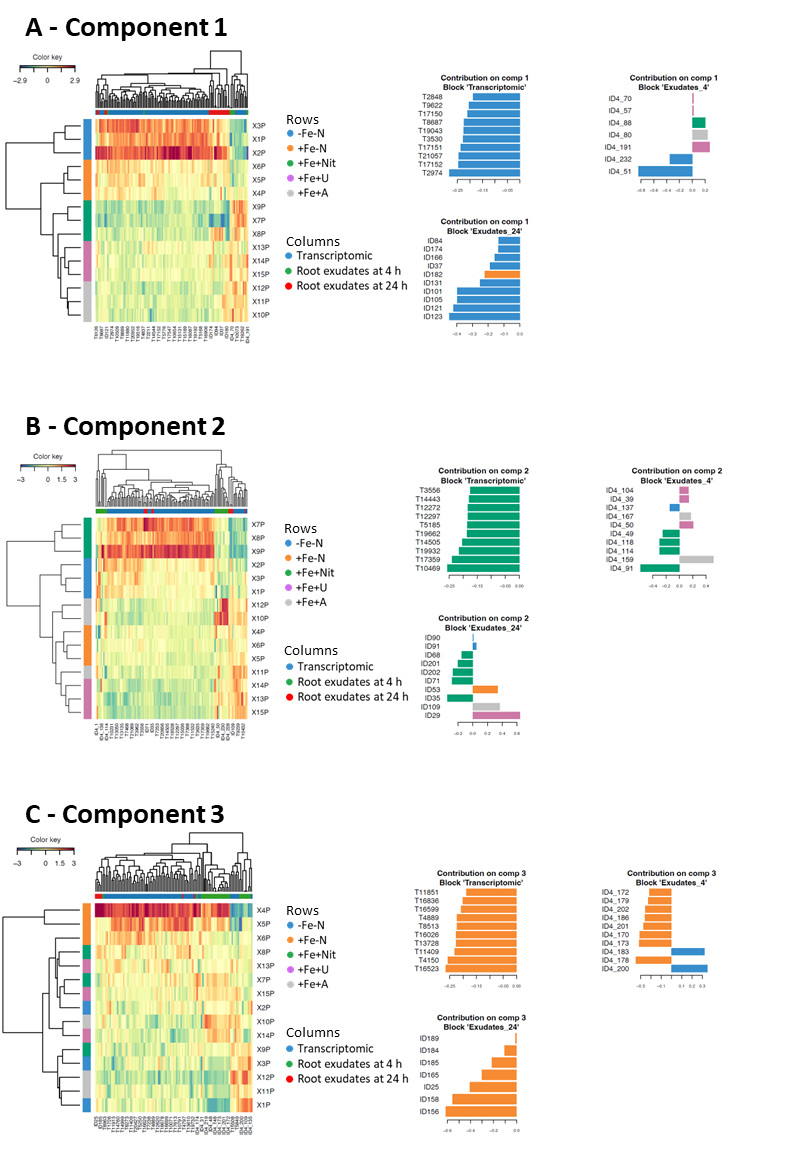


**Supplementary Figure S6**. Heatmaps of component 1 (A), component 2 (B) and component 3 (C) and bar plots of the contribution of the max. 10 most characterizing “features” (modulated transcript, or metabolic exudate at 4 hours or metabolic exudate at 24 hours) for each component.
